# Supplementary material for: Distinct neural mechanisms for heading retrieval and context recognition in the hippocampus during spatial reorientation
Source: Nat Commun. 2024 Jul 16;15:5968. doi: 10.1038/s41467-024-50112-7 (PMC11252339; doi:10.1038/s41467-024-50112-7)
Supplement: Supplementary file 1 — Supplementary Information [file 41467_2024_50112_MOESM1_ESM.pdf]

Individual digging data per animal

		Context A				Context B			
Animal	Day	Correct	Geo	Near	Far	Correct	Geo	Near	Far
AK42	1	0%	100%	0%	0%	100%	0%	0%	0%
AK74	1	0%	50%	0%	50%	0%	100%	0%	0%
CMG087	1	50%	50%	0%	0%	50%	50%	0%	0%
CMG089	1	50%	0%	0%	50%	0%	100%	0%	0%
CMG129	1	50%	50%	0%	0%	0%	100%	0%	0%
CMG154	1	50%	50%	0%	0%	0%	0%	0%	100%
CMG159	1	0%	50%	50%	0%	50%	50%	0%	0%
CMG161	1	50%	50%	0%	0%	50%	0%	50%	0%
CMG162	1	50%	0%	0%	50%	0%	50%	0%	50%
CMG169	1	50%	0%	50%	0%	100%	0%	0%	0%
HG1	1	100%	0%	0%	0%	50%	50%	0%	0%
JJ9	1	0%	50%	50%	0%	0%	0%	50%	50%
K1	1	50%	50%	0%	0%	0%	50%	50%	0%
MG1	1	50%	50%	0%	0%	50%	50%	0%	0%
AK42	2	50%	17%	33%	0%	33%	17%	17%	33%
AK74	2	33%	17%	17%	33%	67%	17%	0%	17%
CMG087	2	50%	25%	0%	25%	25%	50%	25%	0%
CMG089	2	67%	17%	0%	17%	60%	20%	0%	20%
CMG129	2	50%	0%	0%	50%	50%	25%	25%	0%
CMG154	2	67%	33%	0%	0%	50%	50%	0%	0%
CMG159	2	60%	20%	0%	20%	50%	17%	17%	17%
CMG161	2	25%	25%	50%	0%	50%	25%	25%	0%
CMG162	2	33%	33%	17%	17%	50%	17%	17%	17%
CMG169	2	67%	33%	0%	0%	67%	33%	0%	0%
HG1	2	33%	67%	0%	0%	67%	0%	0%	33%
JJ9	2	33%	17%	50%	0%	67%	17%	17%	0%
K1	2	33%	50%	0%	17%	67%	17%	17%	0%
MG1	2	67%	33%	0%	0%	67%	17%	0%	17%
AK42	3	33%	33%	17%	17%	17%	50%	0%	33%
AK74	3	50%	50%	0%	0%	17%	17%	67%	0%
CMG087	3	33%	17%	50%	0%	33%	50%	0%	17%
CMG089	3	20%	40%	20%	20%	50%	17%	17%	17%
CMG129	3	33%	33%	17%	17%	33%	0%	17%	50%
CMG154	3	0%	50%	0%	50%	33%	67%	0%	0%
CMG159	3	50%	17%	17%	17%	33%	33%	0%	33%
CMG161	3	17%	17%	17%	50%	83%	0%	17%	0%
CMG162	3	83%	0%	17%	0%	50%	33%	17%	0%
CMG169	3	40%	0%	40%	20%	83%	0%	0%	17%
HG1	3	75%	25%	0%	0%	100%	0%	0%	0%
JJ9	3	33%	50%	0%	17%	100%	0%	0%	0%
K1	3	67%	17%	17%	0%	60%	0%	40%	0%
MG1	3	50%	50%	0%	0%	67%	17%	17%	0%

Supplementary Table 1. Complement of Fig. 1. Individual data points corresponding to Fig. 1c showing percent of digs in each cup per context.

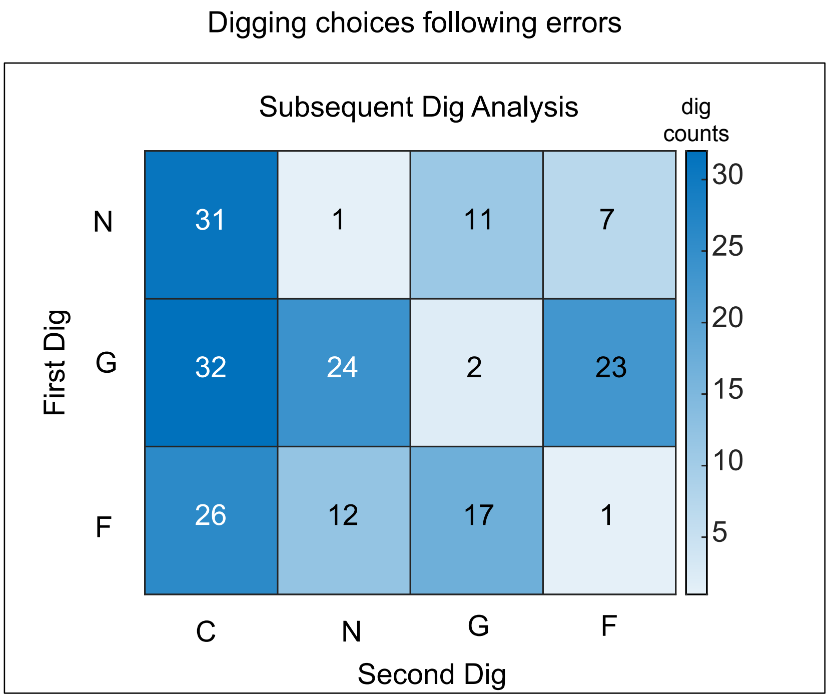

Supplementary Fig. 1. Complement of Fig. 1. Digging counts following errors in Near (N), Geo (G), or Far (F). Following errors in G, N, or F the likelihood that the next dig was Correct (C) was higher than digging in error locations. This is evident in the higher counts observed in column C, depicted in darker blue colors. Following G digs, there was a moderate probability of errors in N or F. Other error combinations were less likely. These data indicate that when animals made errors, they self-corrected going to the C location in most cases. Correct first choices are not shown because following these digs animals stayed in the C cup until the reward was found. Source data are provided as a Source Data file.

Individual and global Bayes Factors (BF) per context based on digging behavior

Condition	Subject	Log (BF)	Condition	Subject	Log (BF)	Condition	Subject	Log (BF)
Context A	AK42	0.6998	Context A	AK42	0.1305	Context A	AK42	0.1305
Day 1	AK74	-0.2400	Day 2	AK74	-0.5651	Day 3	AK74	2.3753
	CMG87	0.6998		CMG87	0.3799		CMG87	-0.5651
	CMG89	-0.2400		CMG89	1.0990		CMG89	-0.1580
	CMG129	0.6998		CMG129	-0.4227		CMG129	0.1305
	CMG154	0.6998		CMG154	1.0879		CMG154	-0.4227
	CMG159	-0.2400		CMG159	0.7277		CMG159	0.1305
	CMG161	0.6998		CMG161	-0.4227		CMG161	-1.0643
	CMG162	-0.2400		CMG162	0.1305		CMG162	1.0990
	CMG169	-0.2400		CMG169	1.0879		CMG169	-0.7839
	HG1	0.6998		HG1	1.0879		HG1	1.4983
	JJ9	-0.2400		JJ9	-0.5651		JJ9	1.0990
	KL1	0.6998		KL1	1.0990		KL1	1.0990
	MG1	0.6998		MG1	2.3753		MG1	2.3753
	Global	**4.1586**		Global	**7.2299**		Global	**6.9434**

Condition	Subject	Log (BF)	Condition	Subject	Log (BF)	Condition	Subject	Log (BF)
Context B	AK42	0.6998	Context B	AK42	-0.5651	Context B	AK42	0.1305
Day 1	AK74	0.6998	Day 2	AK74	1.0990	Day 3	AK74	-1.0643
	CMG87	0.6998		CMG87	0.3799		CMG87	1.0990
	CMG89	0.6998		CMG89	0.7277		CMG89	0.1305
	CMG129	0.6998		CMG129	0.3799		CMG129	-1.0643
	CMG154	-0.5108		CMG154	1.4983		CMG154	1.0879
	CMG159	0.6998		CMG159	0.1305		CMG159	0.1305
	CMG161	-0.2400		CMG161	0.3799		CMG161	1.0990
	CMG162	-0.2400		CMG162	0.1305		CMG162	1.0990
	CMG169	0.6998		CMG169	1.0879		CMG169	1.0990
	HG1	0.6998		HG1	0.0570		HG1	1.4983
	JJ9	-0.8835		JJ9	1.0990		JJ9	2.3753
	KL1	-0.2400		KL1	1.0990		KL1	-0.1580
	MG1	0.6998		MG1	1.0990		MG1	1.0990
	Global	**4.1839**		Global	**8.6024**		Global	**8.5614**

Supplementary Table 2. Complement of Fig. 1f. Individual and global Bayes Factors (BF) to test the alternative model (M_{Alt}) that animals preferentially dug on the correct axis in each context (long wall right in context A and long wall left in context B, Fig.1) vs. the null model (M_{null}) that animals dug by chance. Note that the global BF were > than 1.1 across days and contexts, providing support for the M_{Alt}.

Individual and global Bayes Factors (BF) evaluating digging behavior in the correct cup in comparison to others

Condition	Subject	Log (BF)	Condition	Subject	Log (BF)	Condition	Subject	Log (BF)
Day 1	AK42	0.2587	Day 2	AK42	0.0529	Day 3	AK42	-1.3172
	AK74	-1.4664		AK74	1.0563		AK74	-0.7228
	CMG87	0.2587		CMG87	-0.3758		CMG87	-0.7228
	CMG89	-0.7739		CMG89	2.9944		CMG89	-0.4658
	CMG129	-0.7739		CMG129	0.6299		CMG129	-0.7228
	CMG154	-0.4009		CMG154	1.1714		CMG154	-1.4844
	CMG159	-0.7739		CMG159	1.5516		CMG159	0.0529
	CMG161	0.2587		CMG161	-0.3758		CMG161	1.0563
	CMG162	-0.7739		CMG162	0.0529		CMG162	3.8949
	CMG169	1.7795		CMG169	1.8580		CMG169	2.9944
	HG1	1.7795		HG1	0.4350		HG1	5.8906
	JJ9	-1.4664		JJ9	1.0563		JJ9	3.8949
	KL1	-0.7739		KL1	1.0563		KL1	2.9944
	MG1	0.2587		MG1	3.8949		MG1	2.3272
	Global	**-2.6096**		Global	**15.0582**		Global	**17.6698**

Supplementary Table 3. Complement of Fig. 1g**.** Individual and global Bayes Factors (BF) to test the alternative model (M_{Alt}) that animals dug in the correct cup location vs. the null model (M_{null}) that animals dug by chance. [log(BF)> 1.1 provides credibility for $M_{Alt,,}$ log(BF) < -1.1 provides credibility for M_{null}]. The results indicated that the alternative model only had credibility on days 2 and 3, indicating that with experience animals dug in the correct cup more than other.

Example of environmental sampling and sampling statistics

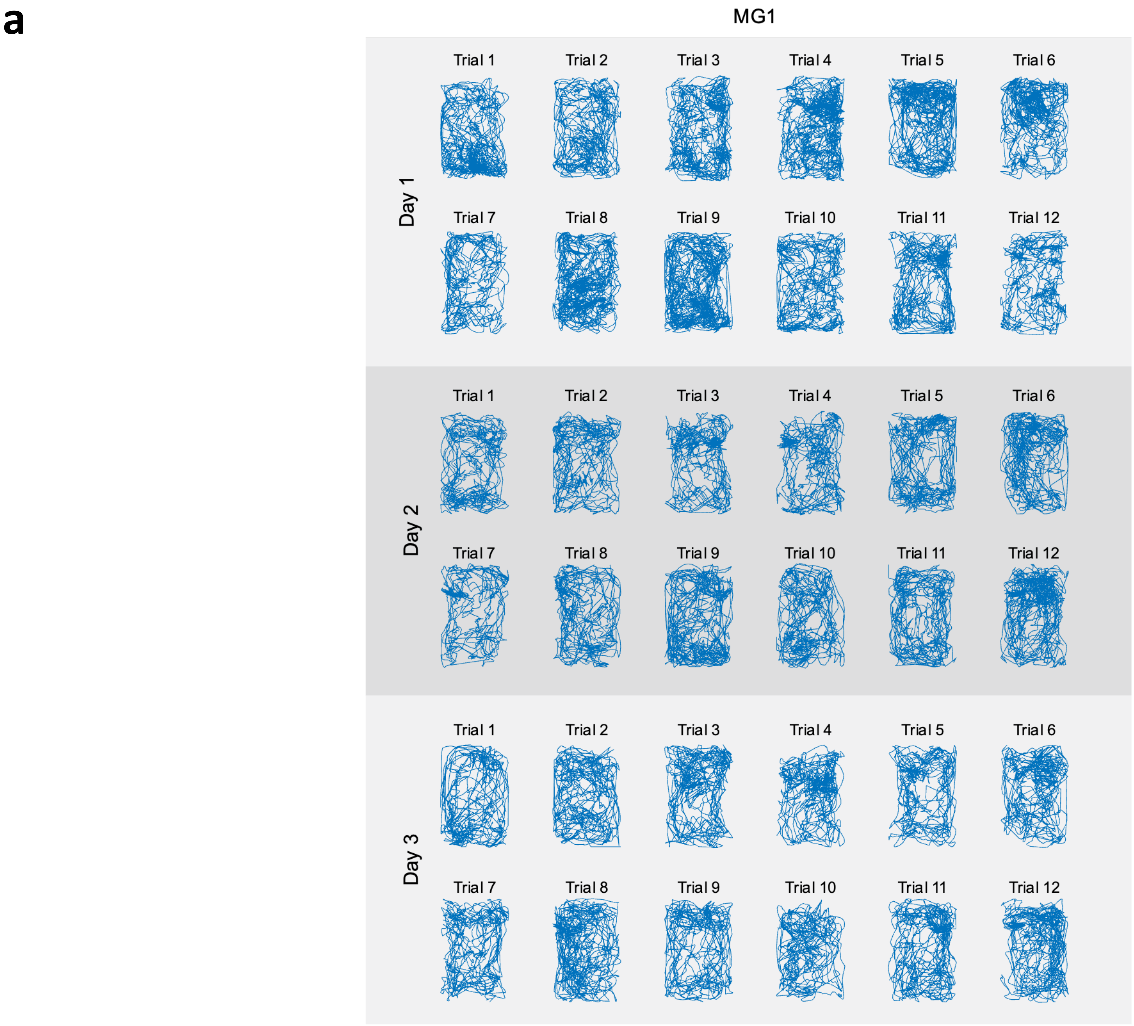

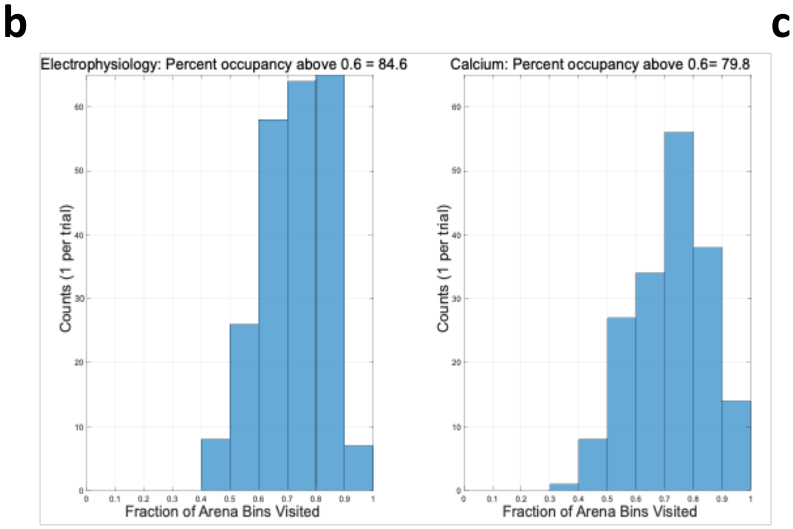

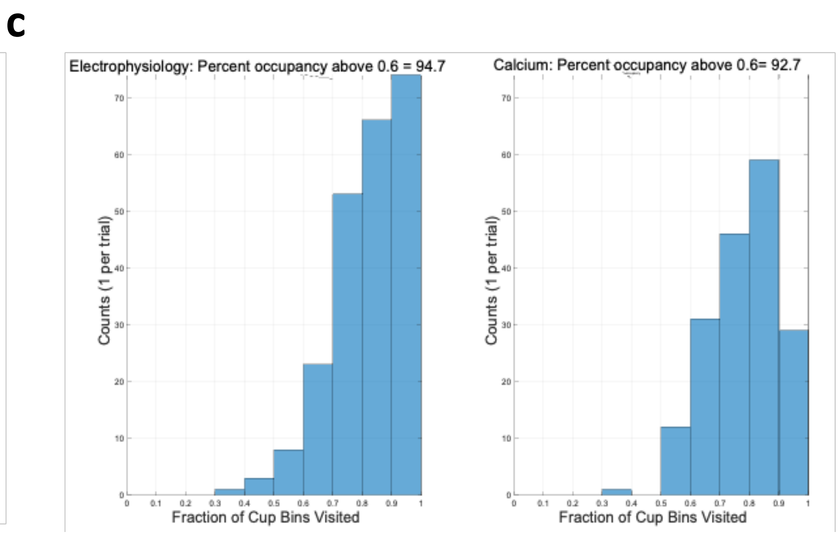

Supplementary Fig. 2. Complement of Fig. 2, 3, 4, 5, 6, and 7. **a)** Sample paths of a mouse (MG1) trained in the two-context task for 3 consecutive days. **b)** Sampling of environments and cups calculated before smoothing. B. Sampling of environments. For electrophysiology and calcium imaging animals, 85% and 81% of the trails included sampling that exceeded 60% of the environment bins, respectively. **c)** Sampling of cups. Cup sampling was calculated by summing the bin occupancy of a circular area over each cup using a 2.25 cm radius. For electrophysiology and calcium imaging animals, 94.7% and 93.1% of the trials included sampling that exceeded 60% of the cup surface bins, respectively. These data show that although the reorientation trials were short, animals displayed appropriate sampling of the environments due to their high motivation to find the reward. Source data are provided as a Source Data file.

Center-out angle method corroborates that place field alignment to spatial geometry persists over days

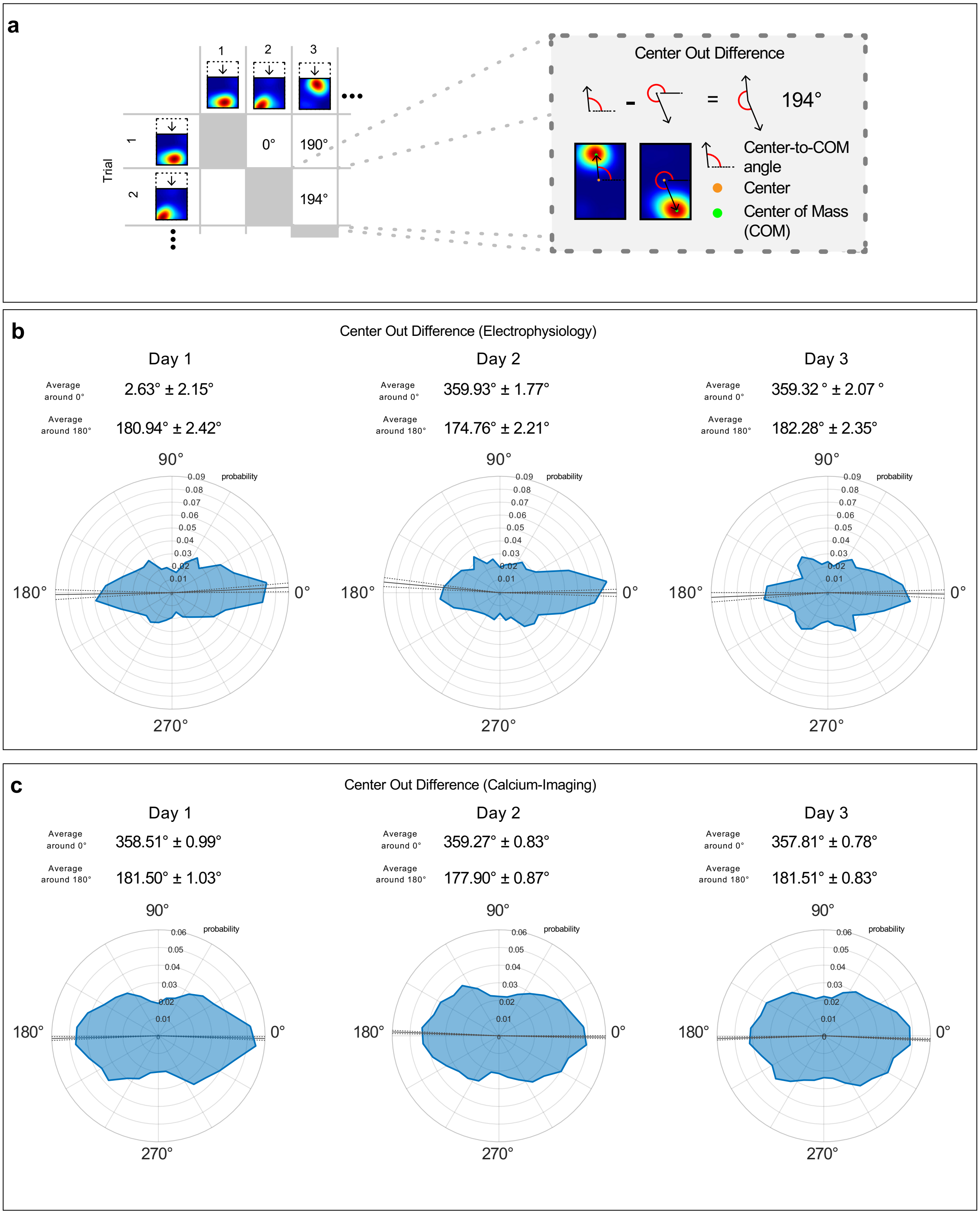

Supplementary Fig. 3. Complement of Fig. 2. Place field alignment to spatial geometry persists over days. **a)** Schematic of center-out angle analysis. For each pairwise comparison, the angle from the center of the map to the center of mass (centroid of the field) is calculated from both maps, and the difference is the center-out difference. **b)** Polar histogram of angle differences from all pairwise comparisons between place cell maps calculated from electrophysiological data. **c)** Polar histogram of center-out angle differences from all pairwise comparisons between place cell maps calculated from calcium imaging data. Note that both electrophysiology and calcium imaging polar plots show peaks that are relatively close to 0º and 180º. Peaks +/- SEM are shown on top of each plot and illustrated on the plots with black and dotted lines, respectively. Note that these values were calculated on the raw data and the plots show binned data using 6º angles. Source data are provided as a Source Data file.

Error prediction using the map orientation and proportion of dig sequences per trial as a function of place field angular rotation

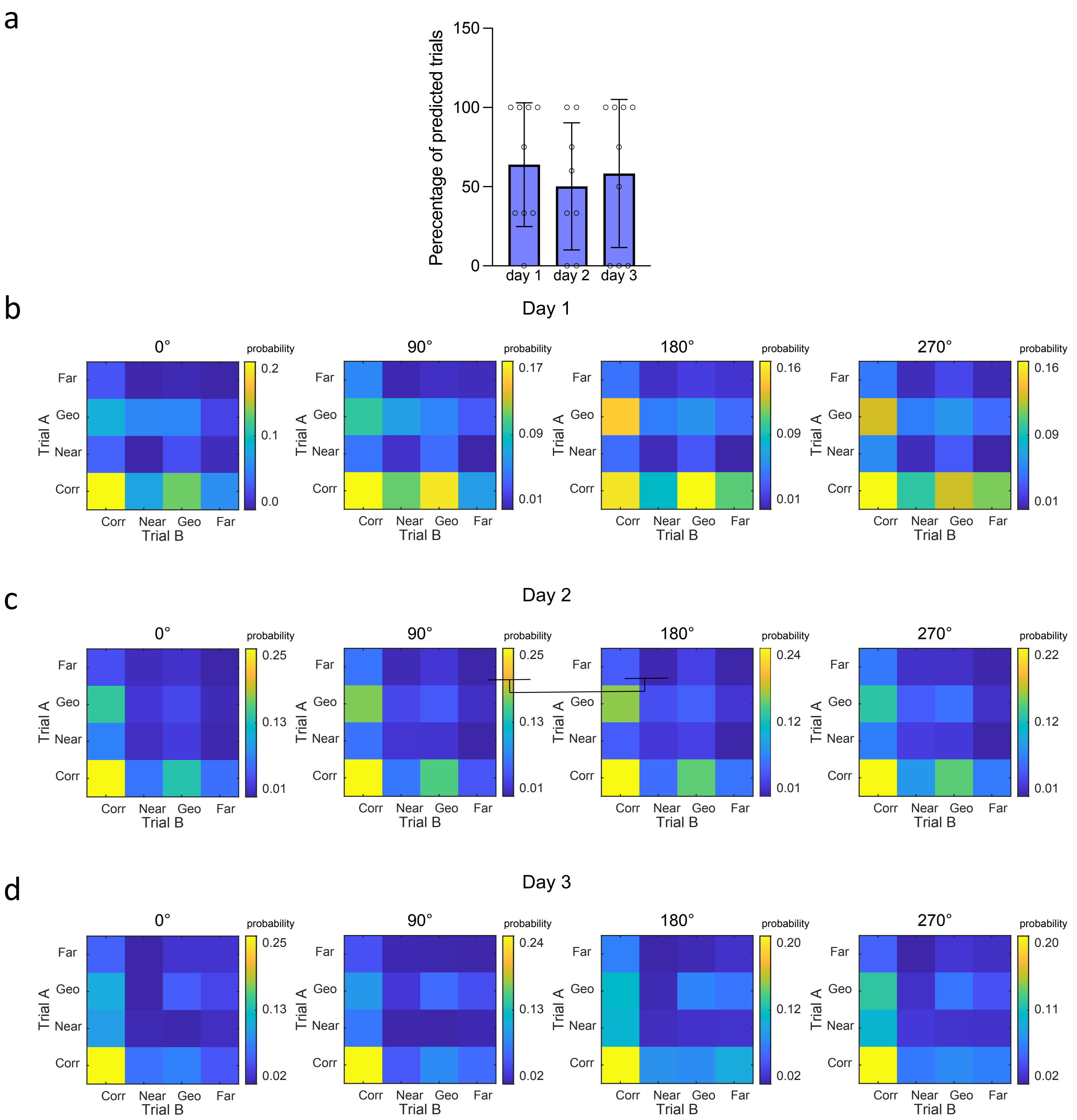

Supplementary Fig. 4. Complement of Fig. 2. Error prediction and proportion of dig sequences per trial as a function of place field angular rotation. **a)** Prediction of N and F error trails using the center out angle of these trials. The sine and cosine of each cells' N and F trials' center-out angle were used to train a support vector machine classifier, validated using a leave-one-out approach. Error trials could not be predicted above chance one sample, robust, one-sample, one-tailed t-test: (Day 1: $\xi = 0.34$, $p = .2800$; Day 2: $\xi = 0.001$, $p = .9800$; Day 3: $\xi = 0.15$, $p = .6080$). **b-d)** Heatmap matrices showing proportion of dig sequences per trial as a function of place field angular rotation (0°, 90°, 180°, 270°) on day 1 (b), day 2 (c), and day 3 (d). Note that C/C and C/G are the most prevalent dig sequences, which is illustrated in vivid yellow/green colors. 90° and 270° rotations happen sporadically, even during C/C G/C or C/G sequences, which may reflect inherent noise in neural data. Colors indicate response counts, with yellow reflecting higher counts and dark blue lower ones.

Modeling approach for the selection of the remapping threshold

a

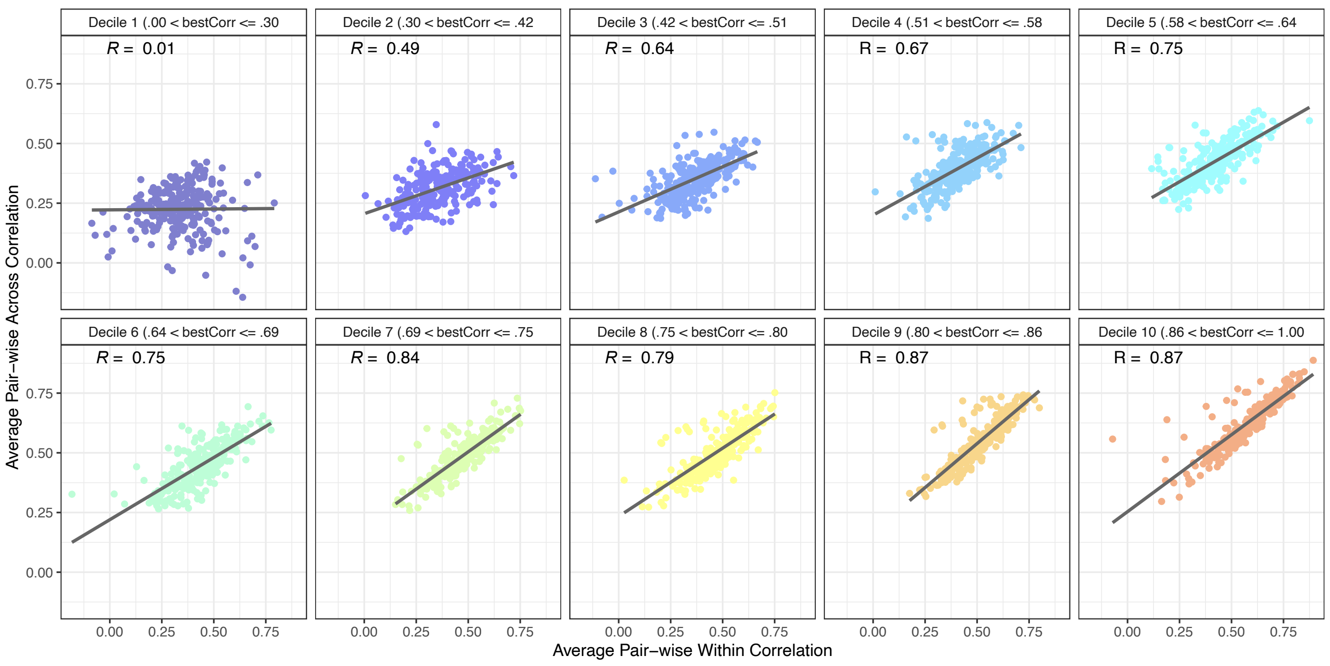

b

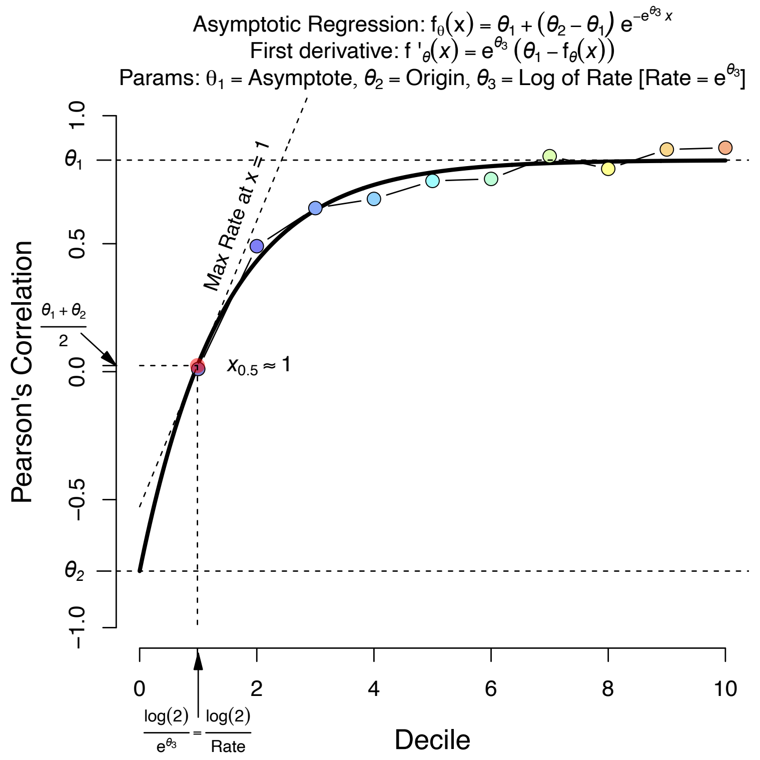

Supplementary Fig. 5. Complement of Fig. 3. Validation of remapping threshold. **a)** Scatterplots showing average correlations within and across context for individual cells separated in deciles obtained from the similarity distribution shown in Figure 3c. **b)** Asymptotic regression model of the overall correlation decile function. The red dot indicates the half life of the function, which coincides with decile 1 (correlations across context between 0 and 0.3) and the root of the function (value that makes the function 0 on the y axis). Finally, in the asymptotic regression model, the relative growth rate is not constant. It attains its peak when $Y = 0$ and diminishes as Y increases. This suggests that Decile 1, corresponding to $Y = 0$, represents the point at which the rate of change is maximized. This indicates that that the first decile is the most informative to discriminate across context (Modeling method detailed in Supplementary Fig. 6 and Supplementary Table 4). Cells included in this analysis had place fields in at least 2 trials in each context to obtain within and across correlation values (n=2515). Source data are provided as a Source Data file.

Schematic showing the models tested to validate the selection of the remapping threshold

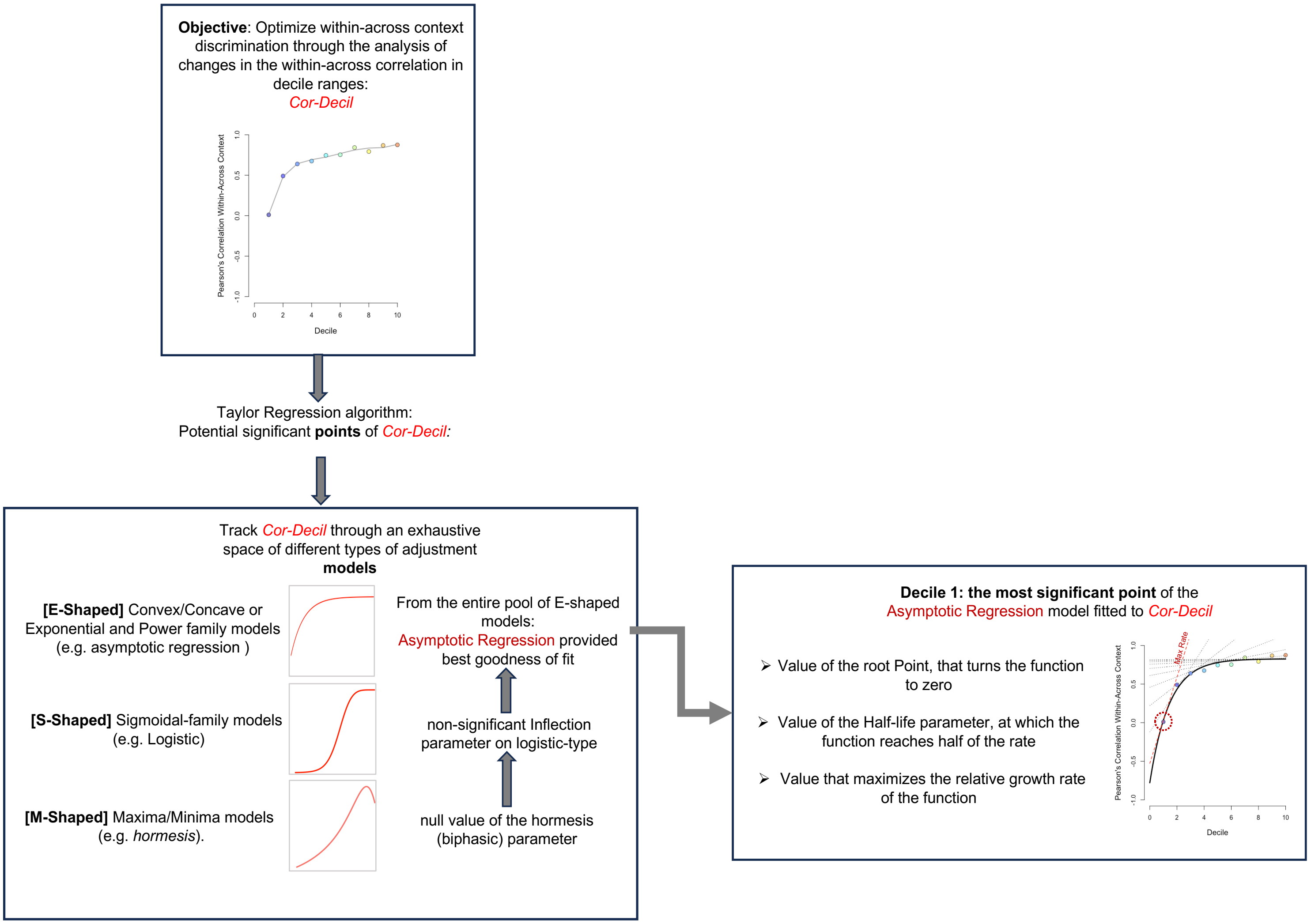

Supplementary Fig. 6. Complement of Fig. 3. To select the best correlation threshold to characterize similarity scores, we first separated the cells into deciles using context similarity distribution shown in Figure 3B For each cell, we plotted the average within and across context correlation (average correlations included all pairwise comparisons corresponding to each cell) and generated scatterplots for each decile (Figure S4A). We then calculated a Pearson's correlation coefficient between the average within and average across correlations for the associated cells per decile, which yielded a set of 10 values (one per decile) (hereafter, named Cor-Decil function). We noted that the largest difference occurred between the first and second decile. To understand this change, we used a modeling approach to fit the data (Supplementary Fig. 4b).

To identify the optimal non-linear model, a two-stage goodness-of-fit analysis was conducted. First, significant points (Roots, Extremes, and Inflections) were pinpointed in the correlation decile function shown in Supplementary Fig. 5b using the Taylor Regression algorithm [1]. Root values denote the X-axis values leading to null Y-axis values, while inflection points, maxima, and minima were determined through derivatives of the function. Second, the best model was selected from an exhaustive pool of alternatives based on the lowest values for Akaike's information criterion (AIC), Residual Standard Error (RSE), and the highest value for log likelihood (L-L) a set of metrics gauging goodness of fit [2]. Notably, Sigmoidal (based on inflection points) and type Maxima/Minima models were excluded due to their inability to yield a satisfactory fit for the data. It is important to note that similar modeling approaches have been previously applied to characterize dynamics of learning curves [3], psychophysics [4], biological processes [5], and distinct neuronal cell types [6].

Key parameters in model selection

Parameter	Estimate	*sd*	*t*	*Pr*(>|*t*|)
Asymptote	0.8300	0.02	34.84	0.0000***
Origin	-0.7800	0.21	-3.65	0.0082**
Rate (Log)	-0.3500	0.15	-2.37	0.0497*

Note: $*** p \leq .001, ** p \leq .01, * p \leq .05$

R^2 = 0.973, RSE= 0.0484, L-L= 17.88, AIC = -27.75

Supplementary Table 4. Complement of Fig. 3. The previous analysis led us to focus on concave-convex or exponential-type models, from which the Asymptotic Regression models (Pinheiro and Bates, 2000) exhibited the best results in terms of goodness of fit [*Akaike's information criterion (AIC)* = -27.75, *Residual Standard Error (RSE)* = .0484, *log likelihood (L-L)* = 17.88].

Asymptotic Regression model: Correlation = $q_1 + (q_2 - q_1) \cdot \exp(-\exp(q_3) \cdot Decile)$.

In this model, q_1: Asymptote, q_2: Origin, and q_3: Rate (on a logarithmic scale).

The term "Origin" refers to the initial correlation value, while the "Asymptote" signifies the point at which correlations stabilize. The "Rate" parameter captures the speed of the correlation function change from the origin to the asymptote, computed as the natural logarithm of the rate constant. The estimated values for the three parameters were all statistically significant (Asymptote = 0.83, Origin = -0.78, LogRate = -0.35; $p < .05$). We then estimated the half-life [$X_{0.5} = \log 2/\mathrm{Rate} = 0.99 \approx 1$, see Figure S4B], which identified the correlation value that reached half of the function's rate. The concept of half-life, a parameter commonly used to characterize exponential changes, is detailed in [7]). In exponential models, the half-life plays a role analogous to the inflection point in sigmoidal (logarithmic) models. In the Correlation-Decile function, Decile 1, comprising correlation values between 0 and 0.3, corresponds to the function's half-life. Additionally, Decile 1 also corresponds to the root of the polynomial function, which is the value that turns the function to zero. Finally, the relative growth rate in the asymptotic regression model (first derivative definition is shown on Figure S4B) is not constant, reaching its maximum value when Y = 0 and decreasing as Y increases. This suggests that Decile 1, corresponding to $Y = 0$, represents the point at which the rate of change is maximized. These results suggest that the optimal correlation threshold value to separate cells based on remapping properties is 0.3.

Proportions of feature insensitive and feature sensitive cells in electrophysiology and calcium imaging recordings

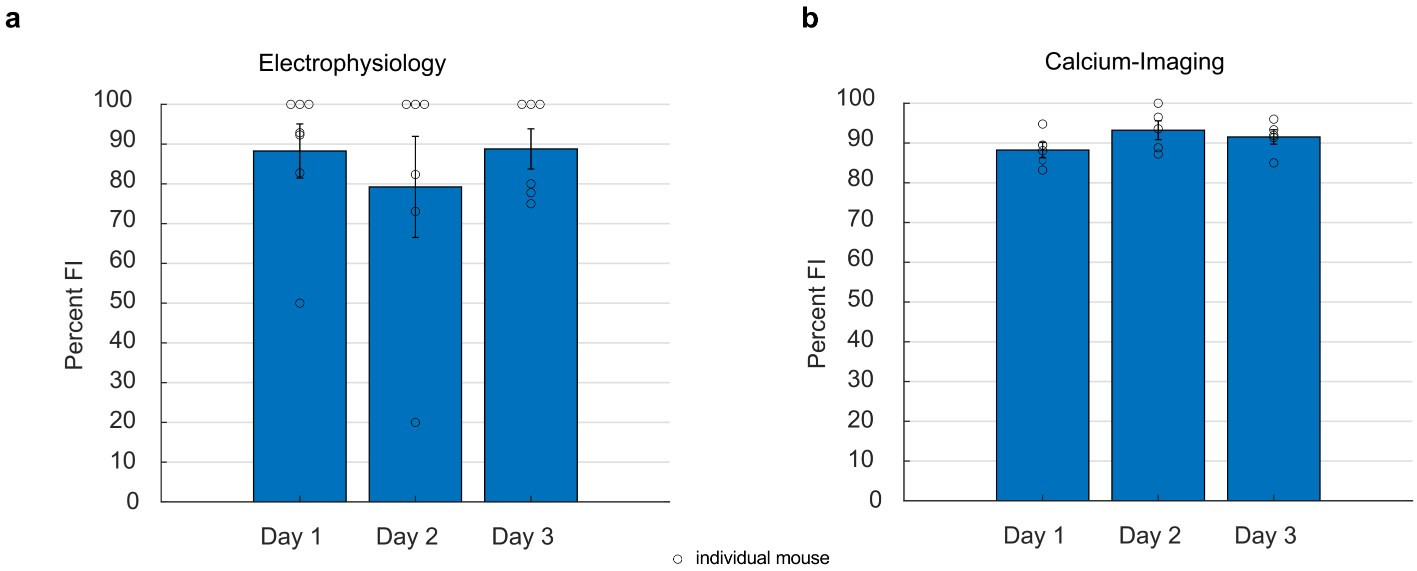

Supplementary Fig. 7. Complement of Fig. 3. Similar proportions of feature-insensitive (FI) cells cells are present in electrophysiology and calcium imaging recordings. **a)** Bar charts showing proportion of FI cells from electrophysiology data (*N*=7 day 1, *N*=6 days 2 and 3). **b)** Bar charts showing proportion of FI cells from calcium-imaging data (*N*=5 all days). Bar charts represent mean ± SEM, circles represent individual data points. Source data are provided as a Source Data file.

Unique Feature-Sensitive cell showing stability in each context

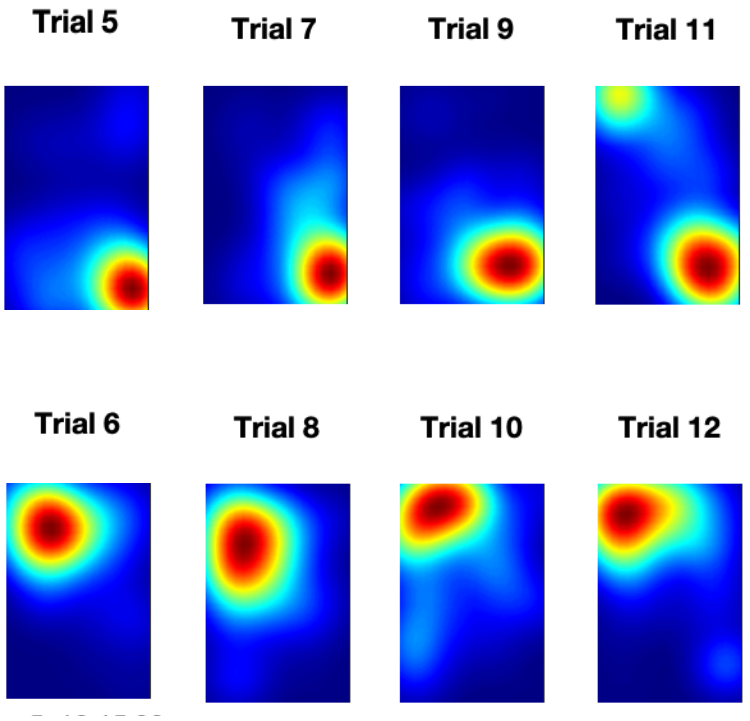

Supplementary Fig. 8. Complement of Fig. 3. The place cell maps shown above correspond to a cell identified among 2669 cells. This cell exhibited high stability in each context but underwent a 180° rotation between contexts (animal JJ9, tetrode 5, cell 4). While this cell serves as an illustration of a perfect FS cell that could have been misclassified by our alignment procedure, no other cells displayed this consistent pattern (we used the sequence of rotations generated by our alignment method to identify any cell that showed stability in one or both contexts, finding only the cell shown above). Notably, this specific cell was recorded with tetrodes on Day 1, and similar cells were not observed on days 2 or 3 in the same animal. Consequently, we concluded that this pattern of results was not representative of our dataset. Trials 1 to 4, which included a visible reward, were excluded from the analysis.

Spatial information content

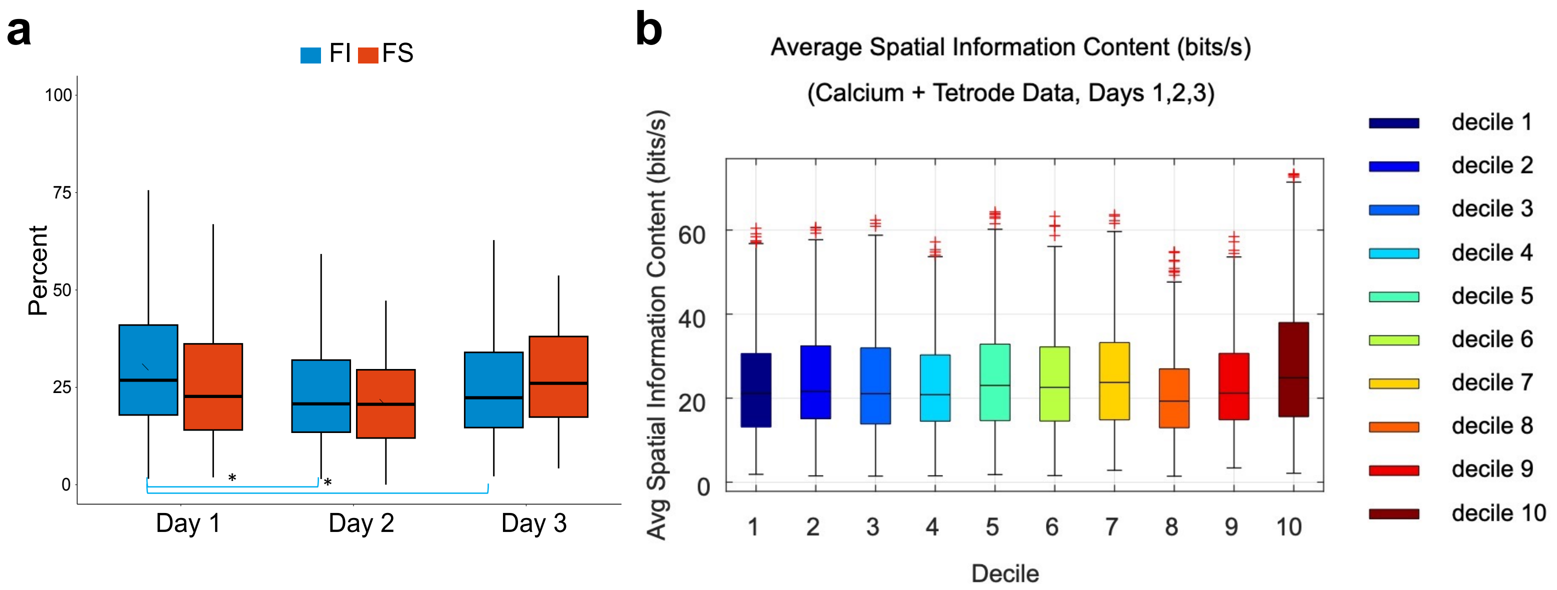

Supplementary Fig. 9. Complement of Fig. 3. Properties of FI and FS cells. **a)** Spatial information content in FS and FI cells. A robust 2-way ANOVA was conducted on spatial information content, with day of testing (day 1 to 3) and Cell type (FI and FS) as between' factors. Results revealed a main effect of day [$F_w(2) = 15.57$, $p = .001$, $\xi = 0.17$], as well as an interaction between day and stability [$F_w(2) = 6.56$, $p = .044$, $\xi = 0.17$], but no effect of Cell Type [$F < 1$]. Robust Rom's multiple comparisons indicated that there were no differences between FI and FS on any testing day ($p > .05$). However, FI cells displayed a difference between days 1 and 2 [$p < .001$; $\xi = 0.26$] and days 1 and 3 [$p < .001$ $\xi = 0.19$], with no differences between days 2 and 3 [$p > .05$; $\xi = 0.08$]. FS cells did not show differences across days ($p > .05$). **b)** Spatial information content grouping data based on similarity correlation values across context divided in deciles (Figure S5). Spatial information content was calculated per day and averaged across days. A one-way ANOVA indicated no differences across deciles [$F(9, 2516) = 1.07$, $p = 0.38$]. The Boxplots boxes indicate the upper and lower quartiles of the data and the whiskers (extending lines) the minimum and maximum outside the quartiles. The horizontal line indicates the median. Total number of cells: *N*=2526. Asterisks (*) represent a significance value set at .05. Red crosses in panel B indicate outliers. Source data are provided as a Source Data file.

Spatial information from calcium traces was calculated using the method developed for calcium traces [8], based on [9]. Spatial information was calculated using the following formula:

$$IC = \sum_{i=1}^{N} {}_{i}\, p_x (x_i) \log (f_i / f)$$

Where f is the mean change in fluorescence, f_i is the mean change in fluorescence in a bin, $p_X(x_i)$ is the probability that the animal is in the ith spatial bin during a time sample. For each cell, we made 2 matrices of binned data, one for the probability of the animal position, p[*x,y*], and one for the binned calcium trace, f [*x,y*]. Both maps were smoothed. We then computed the equation shown above, which gave one spatial information value per cell.

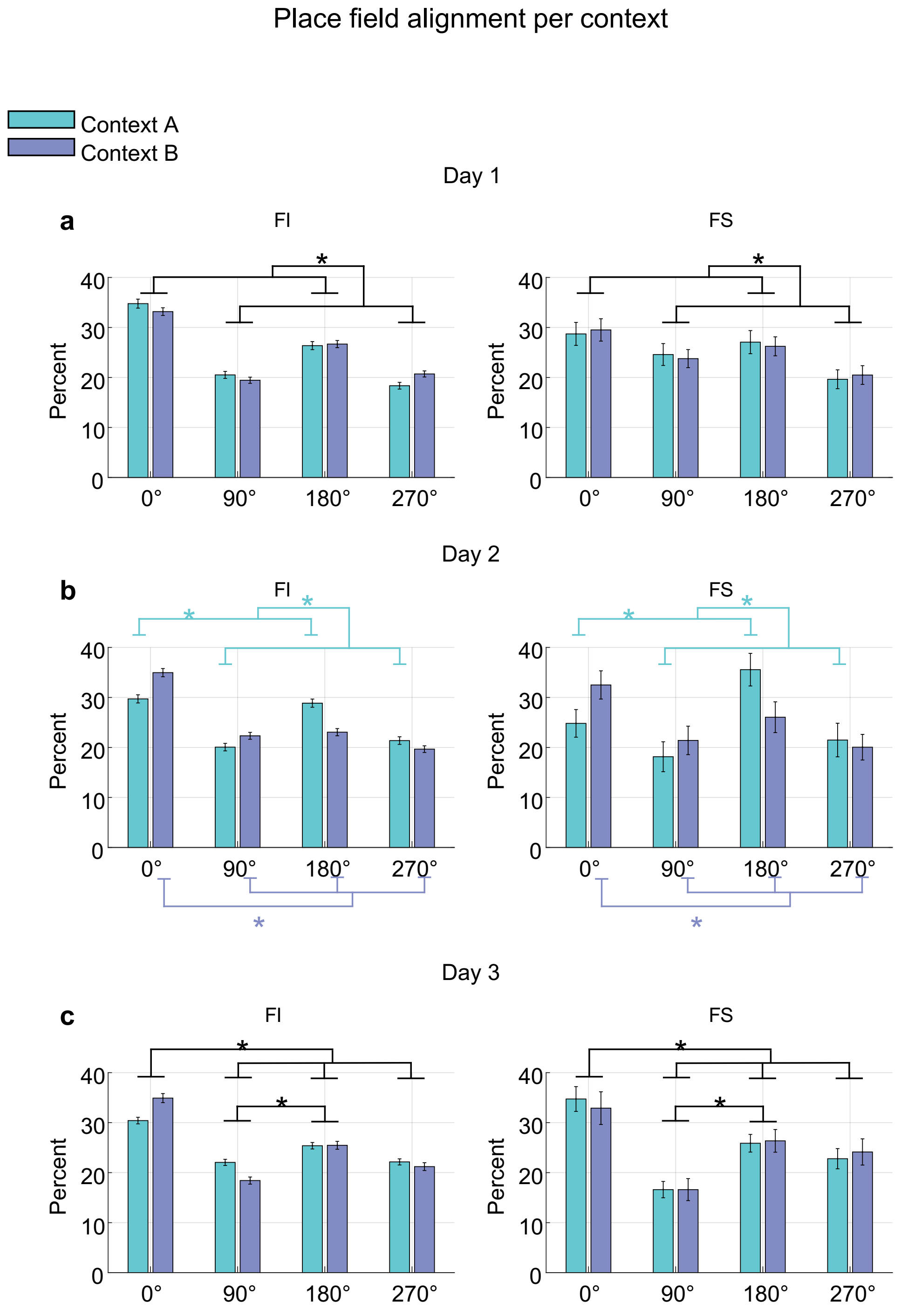

Supplementary Fig. 10. Complement of Fig. 3. Bar charts showing that FI and FS cells display similar geometric alignment within context. **a-c)** Our analysis of map similarity across context indicated that FS cells displayed feature-sensitive remapping across context. However, we observed that when analyzed as a group, without distinguishing between FS and FI, the population of active cells aligned to geometry (Figure 3c-d). This result could happen if both FS and FI cells align to geometry within context despite having different remapping characteristics across context. To investigate this possibility, we evaluated the alignment of FS and FI within each context by conducting the best match rotation analysis depicted in Figure 3a, but this time broken down by chamber. This analysis was performed on the calcium imaging data to have sufficient data points for FS cells. A 4-way repeated measures ANOVA with Context (A or B) and Rotation (0°, 90°, 180°, 270°) as within cells' factors, and cell type (FI or FS) and day (days 1-3) as between factors showed both FI and FS cells displayed geometric alignment within each context. There was a significant main effect of rotation [$F(2.89, 5827.38) = 78.27$, $p < .001$. Additionally, there was a significant interaction between Day, Context and Rotation [$F(5.83, 5884.64) = 2.86$, $p < .001$; the complete results of this ANOVA are shown in Table S5]. In agreement with our hypothesis, post hoc Rom's tests showed that the best match rotations at 0° and 180° occurred more often than other rotations in both Context A and Context B ($p < .05$ on days 1 and 3). Even though geometric alignment persisted throughout training, on day 3 both FS and FI cells also showed higher proportion of best match rotations at 0° than 180º in both contexts (panel c, $p < .05$), paralleling the increase in number of correct digs. Bar chart represent mean ± standard error of the mean (SEM). Asterisks (*) represent a significance value set at .05. Source data are provided as a Source Data file.

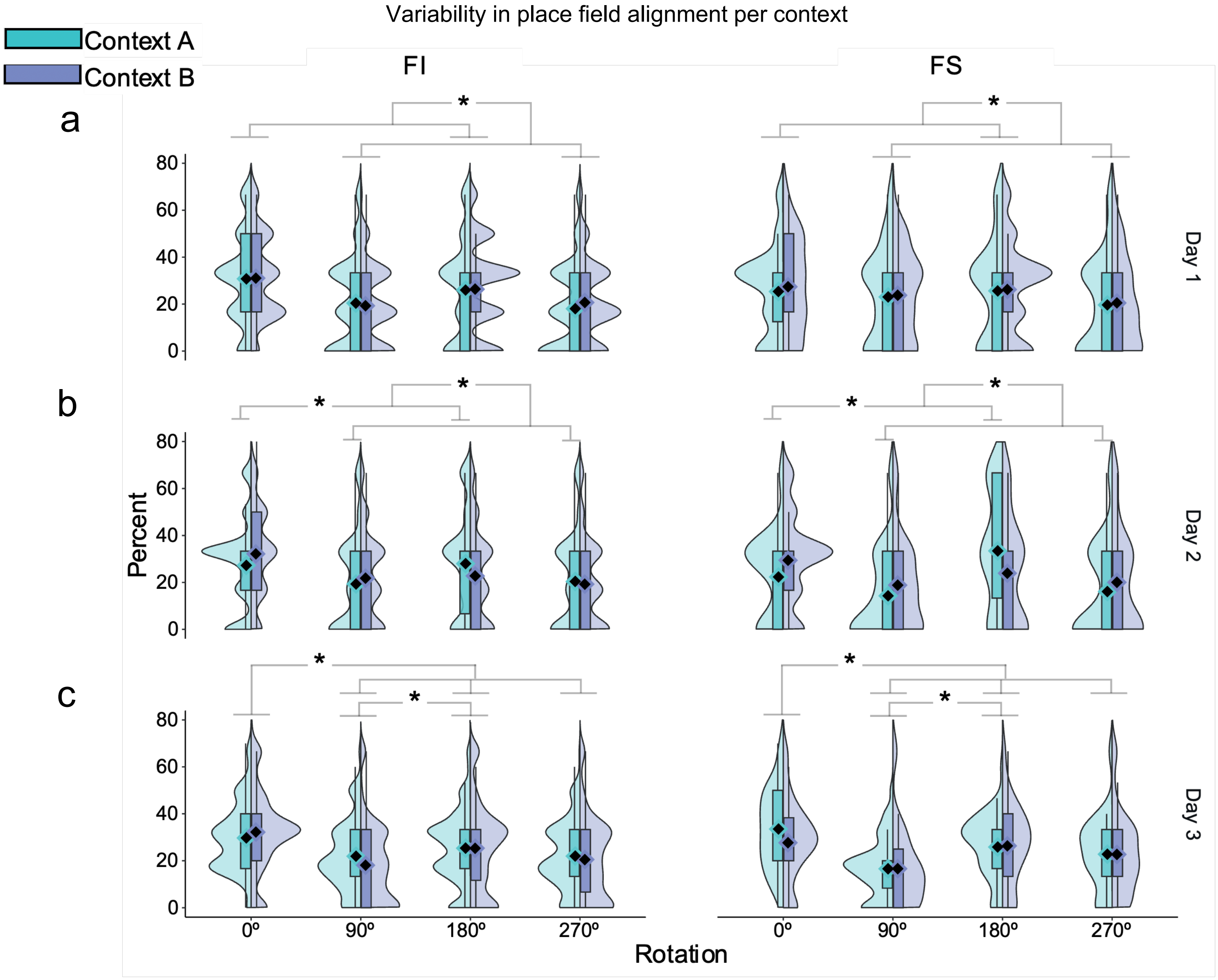

Supplementary Fig. 11. Complement of Fig. 3. **a-c)** Same data shown in Figure S10 plotted as boxplots with overlaid violin plots to depict variability in the data on day 1 (a), day 2 (b), and day 3 (c). Violin plots display a rotated kernel density plot on each side, estimating the density function with kernels as weights. The width represents data density, with peaks indicating frequent values and valleys indicating less frequent ones. Inside the violins, box plots show the median (horizontal line), interquartile range (boxes), variability outside the upper and lower quartiles (whiskers), and a diamond symbol representing the weighted mean used for statistics. Asterisks (*) represent a significance value set at .05. Source data are provided as a Source Data file.

Statistical details about place field alignment per context in FI and FS cells

Predictor	df_{Num}	df_{Den}	*Epsilon*	SS_{Num}	SS_{Den}	*F*	η^2_g	*p*
Cell-type	1.00	2018.00		0.00	0.00	0.00	<.001	.995
Day	2.00	2018.00		0.00	0.00	0.01	<.001	.995
Context	1.00	2018.00		0.00	0.00	0.00	<.001	>.999
Rotation	2.89	5827.38	0.96	11.31	291.37	78.27***	.019	<.001
Cell-type x Day	2.00	2018.00		0.00	0.00	0.00	<.001	.999
Cell-type x Context	1.00	2018.00		0.00	0.00	0.00	<.001	.997
Day x Context	2.00	2018.00		0.00	0.00	0.00	<.001	.998
Cell-type x Rotation	2.89	5827.38	0.96	0.27	291.37	1.85	<.001	.138
Day x Rotation	5.78	5827.38	0.96	0.55	291.37	1.90	<.001	.080
Context x Rotation	2.92	5884.64	0.97	0.45	294.23	3.11*	<.001	.027
Cell-type x Day x Context	2.00	2018.00		0.00	0.00	0.00	<.001	>.999
Cell-type x Day x Rotation	5.78	5827.38	0.96	0.55	291.37	1.91	<.001	.079
Cell-type x Context x Rotation	2.92	5884.64	0.97	0.09	294.23	0.59	<.001	.619
Day x Context x Rotation	5.83	5884.64	0.97	0.83	294.23	2.86**	.001	.009
Cell-type x Day x Context x Rotation	5.83	5884.64	0.97	0.20	294.23	0.70	<.001	.645

Supplementary Table 5. Complement of Fig. 3. Statistical Table corresponding to Supplementary Fig. 10-11. A 4-way repeated measures ANOVA with Context (A or B) and Rotation (0°, 90°, 180°, 270°) as within-cells' factors, and Cell Type (Feature-Sensitive or Feature-Insensitive) and Day (Day 1 to 3) as between factors was conducted to determine if the pattern of alignment in each context was similar in FS and FI cells. df_{Num} indicates degrees of freedom numerator. df_{Den} indicates degrees of freedom denominator. Epsilon indicates Greenhouse-Geisser multiplier for degrees of freedom, p-values and degrees of freedom in the table incorporate this correction. SS_{Num} indicates sum of squares numerator. SS_{Den} indicates sum of squares denominator. η^2_g indicates generalized eta-squared. * $p < .05$, ** $p < .01$, *** $p < .001$.

a

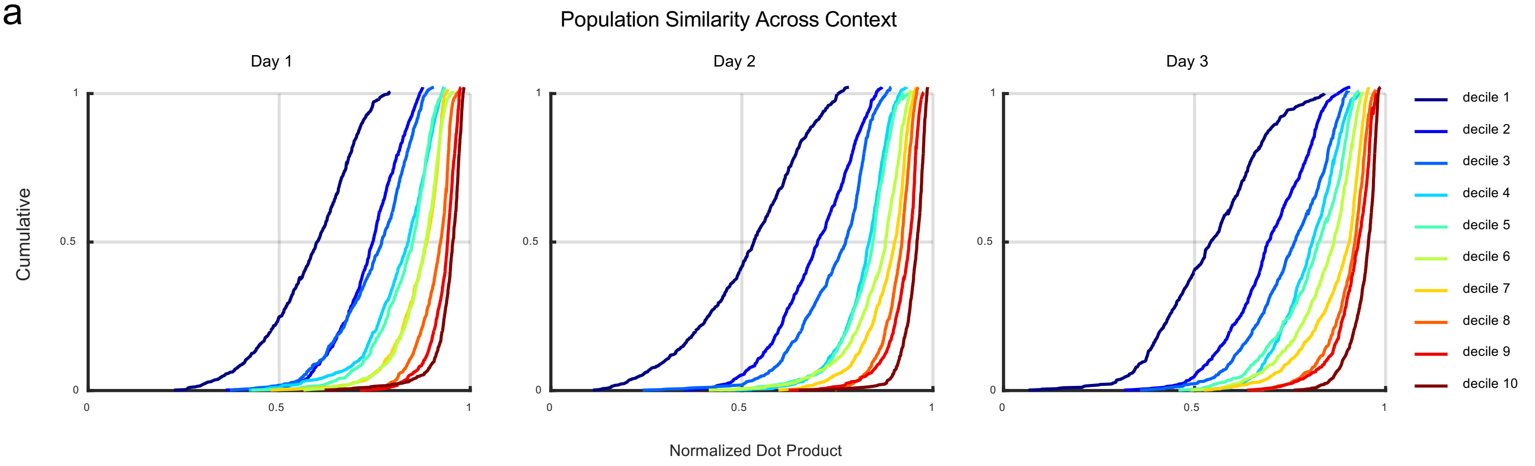

b

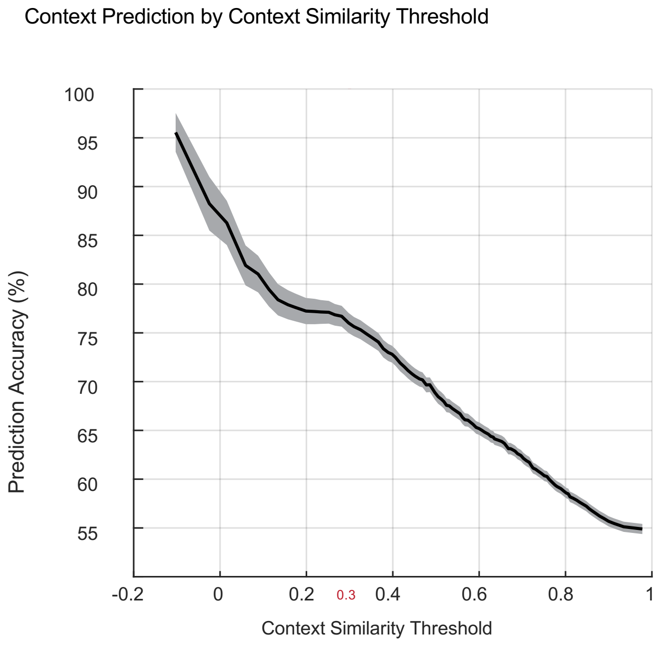

Supplementary Fig. 12. Complement of Fig. 4. **a)** Population similarity across context represented by cumulative proportions. Average aligned maps recorded in Context A and B were stacked and the normalized dot product was computed between population vectors in each corresponding pixel across context. The cells were divided by deciles obtained from the average distribution of correlations across context shown in Fig. 2C. Although the cumulative distributions of dot products display a graded pattern that increases with decile, the cumulative curve corresponding to decile 1 is markedly shifted to the left in comparison to the other distributions. This suggests that cells in decile 1 show lower population similarity across context than cells in the other deciles. **b)** Context prediction as a function of remapping threshold across context. Context prediction decreases as thresholds values increase. However, there is a plateau between 0.2-0.3 correlation values, and a substantial decline at higher correlation values. Source data are provided as a Source Data file.

Analysis of coherency in feature-insensitive and feature sensitive cells

Condition	ci1	ci2	Mean	test	se	n	Effect Size	Size	EfSize.ci1	EfSize.ci2	p	Significance
D1.FI.within.C_1	0.34	0.39	0.37	10.73	0.01	51	1.61	Large	1.27	2.12	<.001	Above
D2.FI.within.C_1	0.36	0.39	0.37	16.28	0.01	69	2.10	Large	1.60	2.83	<.001	Above
D3.FI.within.C_1	0.36	0.39	0.38	17.08	0.01	124	1.64	Large	1.29	1.97	<.001	Above
D1.FS.within.C_1	0.40	0.46	0.43	11.53	0.02	51	1.73	Large	1.23	2.96	<.001	Above
D2.FS.within.C_1	0.41	0.47	0.44	12.56	0.02	57	1.78	Large	1.36	3.36	<.001	Above
D3.FS.within.C_1	0.49	0.63	0.56	8.83	0.04	124	0.85	Large	0.74	1.38	<.001	Above
D1.FI.across.C_1	0.34	0.38	0.36	9.43	0.01	67	1.23	Large	1.09	2.00	<.001	Above
D2.FI.across.C_1	0.34	0.37	0.36	16.63	0.01	89	1.89	Large	1.41	2.60	<.001	Above
D3.FI.across.C_1	0.34	0.36	0.35	20.51	0.00	150	1.79	Large	1.56	2.14	<.001	Above
D1.FS.across.C_1	0.39	0.45	0.42	11.55	0.01	67	1.51	Large	1.07	2.13	<.001	Above
D2.FS.across.C_1	0.43	0.47	0.45	17.41	0.01	73	2.18	Large	1.78	3.48	<.001	Above
D3.FS.across.C_1	0.46	0.59	0.52	8.26	0.03	150	0.72	Medium	0.62	1.32	<.001	Above
D1.FI.within.C_2	0.25	0.28	0.27	2.82	0.01	51	0.42	Small	0.13	0.84	.0084	Above
D2.FI.within.C_2	0.25	0.27	0.26	2.81	0.00	69	0.36	Small	0.10	0.64	.0075	Above
D3.FI.within.C_2	0.26	0.27	0.26	3.73	0.00	124	0.36	Small	0.16	0.59	.0004	Above
D1.FS.within.C_2	0.27	0.3	0.29	4.3	0.01	51	0.64	Medium	0.36	0.94	.0002	Above
D2.FS.within.C_2	0.28	0.3	0.29	7.52	0.01	57	1.07	Large	0.34	2.69	<.001	Above
D3.FS.within.C_2	0.19	0.27	0.23	-1.15	0.02	124	-0.11	Very Small	-0.29	0.07	.2521	Chance
D1.FI.across.C_2	0.26	0.28	0.27	3.23	0.01	67	0.42	Small	0.14	0.77	.0025	Above
D2.FI.across.C_2	0.26	0.27	0.26	4.61	0.00	89	0.52	Medium	0.23	0.73	<.001	Above
D3.FI.across.C_2	0.26	0.27	0.27	7.44	0.00	150	0.65	Medium	0.48	0.78	<.001	Above
D1.FS.across.C_2	0.25	0.28	0.27	2.77	0.01	67	0.36	Small	0.14	0.60	.0085	Above
D2.FS.across.C_2	0.27	0.29	0.28	6.28	0.01	73	0.79	Medium	0.39	3.05	<.001	Above
D3.FS.across.C_2	0.19	0.26	0.22	-1.65	0.02	150	-0.14	Very Small	-0.30	0.01	.1023	Chance

Supplementary Table 6. Complement of Fig. 5E. Best Match rotation (BMR) analysis: Wilcoxon, one-sample, one tailed test with respect to the chance level. First column indicates the experimental condition from the combination of day (D1, D2, and D3, respectively for days 1 to 3), cell type (FI vs. FS), context condition (within vs. across), and BMR (C_1=1st BMR, C_2=2nd BMR). The table indicates that the 1st and 2nd BMR are significantly above chance across days and context condition, except for FS where the 2nd BMR is below chance on day 3, either within or across context. BMR 3 and 4 were below chance across days and contextual conditions for both cell types (data not shown, $p > .05$).

Statistical detail about coherency analysis and marginal probabilities of the 1st and 2nd coherency (best match rotations, BMRs)

a

4-way ANOVA on proportion of consistently rotated cells

Predictor	df_{Num}	df_{Den}	SS_{Num}	SS_{Den}	F	p	η^2_g
(Intercept)	1	1060	224.81	5.37	44400.89	.000	.89
Cell type	1	1060	1.45	5.37	286.72*	.000	.05
Context	1	1060	0.05	5.37	9.01*	.003	.00
Day	2	1060	0.18	5.37	17.62*	.000	.01
BMR Order	1	1060	13.56	23.53	610.81*	.000	.32
Cell type x Context	1	1060	0.01	5.37	1.96	.162	.00
Cell type x Day	2	1060	0.17	5.37	16.94*	.000	.01
Context x Day	2	1060	0.01	5.37	0.80	.450	.00
Cell type x BMR Order	1	1060	1.69	23.53	76.15*	.000	.06
Context x BMR Order	1	1060	0.01	23.53	0.23	.630	.00
Day x BMR Order	2	1060	1.74	23.53	39.30*	.000	.06
Cell type x Context x Day	2	1060	0.00	5.37	0.36	.696	.00
Cell type x Context x BMR Order	1	1060	0.01	23.53	0.36	.551	.00
Cell type x Day x BMR Order	2	1060	1.78	23.53	40.06*	.000	.06
Context x Day x BMR Order	2	1060	0.04	23.53	0.90	.409	.00
Cell type x Context x Day x BMR Order	2	1060	0.00	23.53	0.06	.944	.00

b

Trend Analysis of Cell-type x Day x BMR-Order interaction

Cell = FI & BMR = 1st:

contrast	estimate	*SE*	*df*	*t*	*p*
linear	0.017804	0.0116	964	1.532	.1260
quadratic	0.007707	0.0194	964	0.397	.6918

Cell = FS & BMR = 1st:

contrast	estimate	*SE*	*df*	*t*	*p*
linear	0.163227	0.0117	964	14.004	<.0001
quadratic	-0.041583	0.0219	964	-1.899	.0579

Cell = FI & BMR = 2nd:

contrast	estimate	*SE*	*df*	*t*	*p*
linear	0.000273	0.0116	964	0.024	.9812
quadratic	-0.001815	0.0194	964	-0.093	.9256

Cell = FS & BMR = 2nd:

contrast	estimate	*SE*	*df*	*t*	*p*
linear	-0.078728	0.0117	964	-6.755	<.0001
quadratic	-0.007014	0.0219	964	-0.320	.7488

Results are averaged over the levels of Context

c

Condition	Comp.	pWilcox	Condition	Comp.	pWilcox	Condition	Comp.	pWilcox	Condition	Comp.	pWilcox	Condition	Comp.	pWilcox	Condition	Comp.	pWilcox
Day 1_across_FS	Global	NA	Day 1_across_FI	Global	NA	Day 2_across_FS	Global	NA	Day 2_across_FI	Global	NA	Day 3_across_FS	Global	NA	Day 3_across_FI	Global	NA
Day 1_across_FS	C1.2_C2.1	0.7086	Day 1_across_FI	C1.2_C2.1	0.9761	Day 2_across_FS	C1.2_C2.1	0.1875	Day 2_across_FI	C1.2_C2.1	0.7812	Day 3_across_FS	C1.2_C2.1	0.0502	Day 3_across_FI	C1.2_C2.1	0.9777
Day 1_across_FS	C1.3_C2.1	0.9504	Day 1_across_FI	C1.3_C2.1	0.0290	Day 2_across_FS	C1.3_C2.1	0.9013	Day 2_across_FI	C1.3_C2.1	0.0502	Day 3_across_FS	C1.3_C2.1	0.8214	Day 3_across_FI	C1.3_C2.1	0.0290
Day 1_across_FS	C1.4_C2.1	0.2188	Day 1_across_FI	C1.4_C2.1	0.9761	Day 2_across_FS	C1.4_C2.1	0.8145	Day 2_across_FI	C1.4_C2.1	0.9812	Day 3_across_FS	C1.4_C2.1	0.0312	Day 3_across_FI	C1.4_C2.1	0.9693
Day 1_across_FS	C1.1_C2.2	0.0878	Day 1_across_FI	C1.1_C2.2	0.9864	Day 2_across_FS	C1.1_C2.2	0.1875	Day 2_across_FI	C1.1_C2.2	0.3564	Day 3_across_FS	C1.1_C2.2	0.1006	Day 3_across_FI	C1.1_C2.2	0.0502
Day 1_across_FS	C1.3_C2.2	0.5000	Day 1_across_FI	C1.3_C2.2	0.9882	Day 2_across_FS	C1.3_C2.2	0.9716	Day 2_across_FI	C1.3_C2.2	0.7918	Day 3_across_FS	C1.3_C2.2	0.8285	Day 3_across_FI	C1.3_C2.2	0.9319
Day 1_across_FS	C1.4_C2.2	0.5000	Day 1_across_FI	C1.4_C2.2	0.9915	Day 2_across_FS	C1.4_C2.2	0.3564	Day 2_across_FI	C1.4_C2.2	0.9915	Day 3_across_FS	C1.4_C2.2	0.6054	Day 3_across_FI	C1.4_C2.2	0.9812
Day 1_across_FS	C1.1_C2.3	0.9915	Day 1_across_FI	C1.1_C2.3	0.0520	Day 2_across_FS	C1.1_C2.3	0.9693	Day 2_across_FI	C1.1_C2.3	0.0290	Day 3_across_FS	C1.1_C2.3	0.9716	Day 3_across_FI	C1.1_C2.3	0.0312
Day 1_across_FS	C1.2_C2.3	0.3932	Day 1_across_FI	C1.2_C2.3	0.9915	Day 2_across_FS	C1.2_C2.3	0.0907	Day 2_across_FI	C1.2_C2.3	0.9860	Day 3_across_FS	C1.2_C2.3	0.9122	Day 3_across_FI	C1.2_C2.3	0.8214
Day 1_across_FS	C1.4_C2.3	0.3918	Day 1_across_FI	C1.4_C2.3	0.7951	Day 2_across_FS	C1.4_C2.3	0.3946	Day 2_across_FI	C1.4_C2.3	0.7951	Day 3_across_FS	C1.4_C2.3	0.7069	Day 3_across_FI	C1.4_C2.3	0.9710
Day 1_across_FS	C1.1_C2.4	0.7929	Day 1_across_FI	C1.1_C2.4	0.2940	Day 2_across_FS	C1.1_C2.4	0.9786	Day 2_across_FI	C1.1_C2.4	0.3125	Day 3_across_FS	C1.1_C2.4	0.9328	Day 3_across_FI	C1.1_C2.4	0.7060
Day 1_across_FS	C1.2_C2.4	0.9882	Day 1_across_FI	C1.2_C2.4	0.9915	Day 2_across_FS	C1.2_C2.4	0.9632	Day 2_across_FI	C1.2_C2.4	0.9860	Day 3_across_FS	C1.2_C2.4	0.6436	Day 3_across_FI	C1.2_C2.4	0.9864
Day 1_across_FS	C1.3_C2.4	0.6073	Day 1_across_FI	C1.3_C2.4	0.8019	Day 2_across_FS	C1.3_C2.4	0.9512	Day 2_across_FI	C1.3_C2.4	0.2904	Day 3_across_FS	C1.3_C2.4	0.9853	Day 3_across_FI	C1.3_C2.4	0.8193
**Condition**	**Comp.**	**pWilcox**	**Condition**	**Comp.**	**pWilcox**	**Condition**	**Comp.**	**pWilcox**	**Condition**	**Comp.**	**pWilcox**	**Condition**	**Comp.**	**pWilcox**	**Condition**	**Comp.**	**pWilcox**
Day 1_within_FS	C1.2_C2.1	0.9861	Day 1_within_FI	C1.2_C2.1	0.8849	Day 2_within_FS	C1.2_C2.1	0.9132	Day 2_within_FI	C1.2_C2.1	0.8985	Day 3_within_FS	C1.2_C2.1	0.9504	Day 3_within_FI	C1.2_C2.1	0.7951
Day 1_within_FS	C1.3_C2.1	0.6073	Day 1_within_FI	C1.3_C2.1	0.2113	Day 2_within_FS	C1.3_C2.1	0.0907	Day 2_within_FI	C1.3_C2.1	0.0272	Day 3_within_FS	C1.3_C2.1	0.2481	Day 3_within_FI	C1.3_C2.1	0.0878
Day 1_within_FS	C1.4_C2.1	0.9783	Day 1_within_FI	C1.4_C2.1	0.9861	Day 2_within_FS	C1.4_C2.1	0.9812	Day 2_within_FI	C1.4_C2.1	0.8985	Day 3_within_FS	C1.4_C2.1	0.8438	Day 3_within_FI	C1.4_C2.1	0.9122
Day 1_within_FS	C1.1_C2.2	0.0488	Day 1_within_FI	C1.1_C2.2	0.1346	Day 2_within_FS	C1.1_C2.2	0.5730	Day 2_within_FI	C1.1_C2.2	0.2049	Day 3_within_FS	C1.1_C2.2	0.0290	Day 3_within_FI	C1.1_C2.2	0.2049
Day 1_within_FS	C1.3_C2.2	0.6073	Day 1_within_FI	C1.3_C2.2	0.9783	Day 2_within_FS	C1.3_C2.2	0.5730	Day 2_within_FI	C1.3_C2.2	0.9783	Day 3_within_FS	C1.3_C2.2	0.9152	Day 3_within_FI	C1.3_C2.2	0.9132
Day 1_within_FS	C1.4_C2.2	0.9861	Day 1_within_FI	C1.4_C2.2	0.9915	Day 2_within_FS	C1.4_C2.2	0.9812	Day 2_within_FI	C1.4_C2.2	0.9915	Day 3_within_FS	C1.4_C2.2	0.9860	Day 3_within_FI	C1.4_C2.2	0.9882
Day 1_within_FS	C1.1_C2.3	0.2049	Day 1_within_FI	C1.1_C2.3	0.0672	Day 2_within_FS	C1.1_C2.3	0.0625	Day 2_within_FI	C1.1_C2.3	0.0938	Day 3_within_FS	C1.1_C2.3	0.2940	Day 3_within_FI	C1.1_C2.3	0.0312
Day 1_within_FS	C1.2_C2.3	0.2858	Day 1_within_FI	C1.2_C2.3	0.8849	Day 2_within_FS	C1.2_C2.3	0.7887	Day 2_within_FI	C1.2_C2.3	0.9728	Day 3_within_FS	C1.2_C2.3	0.7086	Day 3_within_FI	C1.2_C2.3	0.9882
Day 1_within_FS	C1.4_C2.3	0.9861	Day 1_within_FI	C1.4_C2.3	0.9783	Day 2_within_FS	C1.4_C2.3	0.9812	Day 2_within_FI	C1.4_C2.3	0.9812	Day 3_within_FS	C1.4_C2.3	0.6068	Day 3_within_FI	C1.4_C2.3	0.9849
Day 1_within_FS	C1.1_C2.4	0.4251	Day 1_within_FI	C1.1_C2.4	0.7146	Day 2_within_FS	C1.1_C2.4	0.1875	Day 2_within_FI	C1.1_C2.4	0.0290	Day 3_within_FS	C1.1_C2.4	0.3125	Day 3_within_FI	C1.1_C2.4	0.0290
Day 1_within_FS	C1.2_C2.4	0.9915	Day 1_within_FI	C1.2_C2.4	0.9915	Day 2_within_FS	C1.2_C2.4	0.9783	Day 2_within_FI	C1.2_C2.4	0.9861	Day 3_within_FS	C1.2_C2.4	0.9728	Day 3_within_FI	C1.2_C2.4	0.9882
Day 1_within_FS	C1.3_C2.4	0.3918	Day 1_within_FI	C1.3_C2.4	0.8849	Day 2_within_FS	C1.3_C2.4	0.5000	Day 2_within_FI	C1.3_C2.4	0.7069	Day 3_within_FS	C1.3_C2.4	0.7086	Day 3_within_FI	C1.3_C2.4	0.9853

Supplementary Table 7: Complement of Fig. 5. Analysis of coherent populations (Fig. 5d and 5e). **a)** Details of 4-way ANOVA on proportion of BMR considering day * cell type (FI vs. FS) * context condition (within vs. across) * BMR-Order (1st vs. 2nd BMR). df_{Num} indicates degrees of freedom numerator. df_{Den} indicates degrees of freedom denominator. SS_{Num} indicates sum of squares numerator. SS_{Den} indicates sum of squares denominator. η^2_g indicates generalized eta-squared. **b)** Trend analysis of Cell-type x Day x BMR-Order interaction. *SE standard error, df degrees of freedom.* Results of ANOVA revealed a main effect of the 4 factors, as well as of the interactions of order 1: Cell-type x Day, Stability x BMR-Order, and Day x BMR-Order; most importantly, a significant interaction between Cell-type x Day x BMR-Order ($p<.05$). Detailed analysis of the significant order 2 interaction was performed using a trend analysis from day 1 to day 3, for each of the Cell-type and BMR-Order combinations. Detailed analysis of tendencies revealed that for FI cells, there is no significant change over days in either BMR 1st or 2nd ($p > .05$ for linear and quadratic). However, in FS cells, a linear change over days is obtained, which is significant for BMR 1st and 2nd ($p<.05$ for linear and $p>.05$ for quadratic), but of opposite sign. * $p < .05$, ** $p <. 01$, *** $p < .001$. BMR: best match rotation. **c)** Results of Wilcoxon test on marginal probabilities shown in Fig. 5F. The First column shows the day of testing, type of contextual comparison (within vs. across), and cell type (FS or FI). The second column shows the best match rotation (BMR) and angular rotation. C1 refers to the 1st BMR and C2 to 2nd BMR. The second number of the comparison refers to angular rotation, 1=0°, 2, 90°, 3=180°, and 4=270°. For instance, C1.1_C2.2 compares the 1st BMR at 0° with the 2nd BMR at 90°. The third column of each condition has the Wilcoxon p level.

Cell registration quality and change in context similarity across days per decile

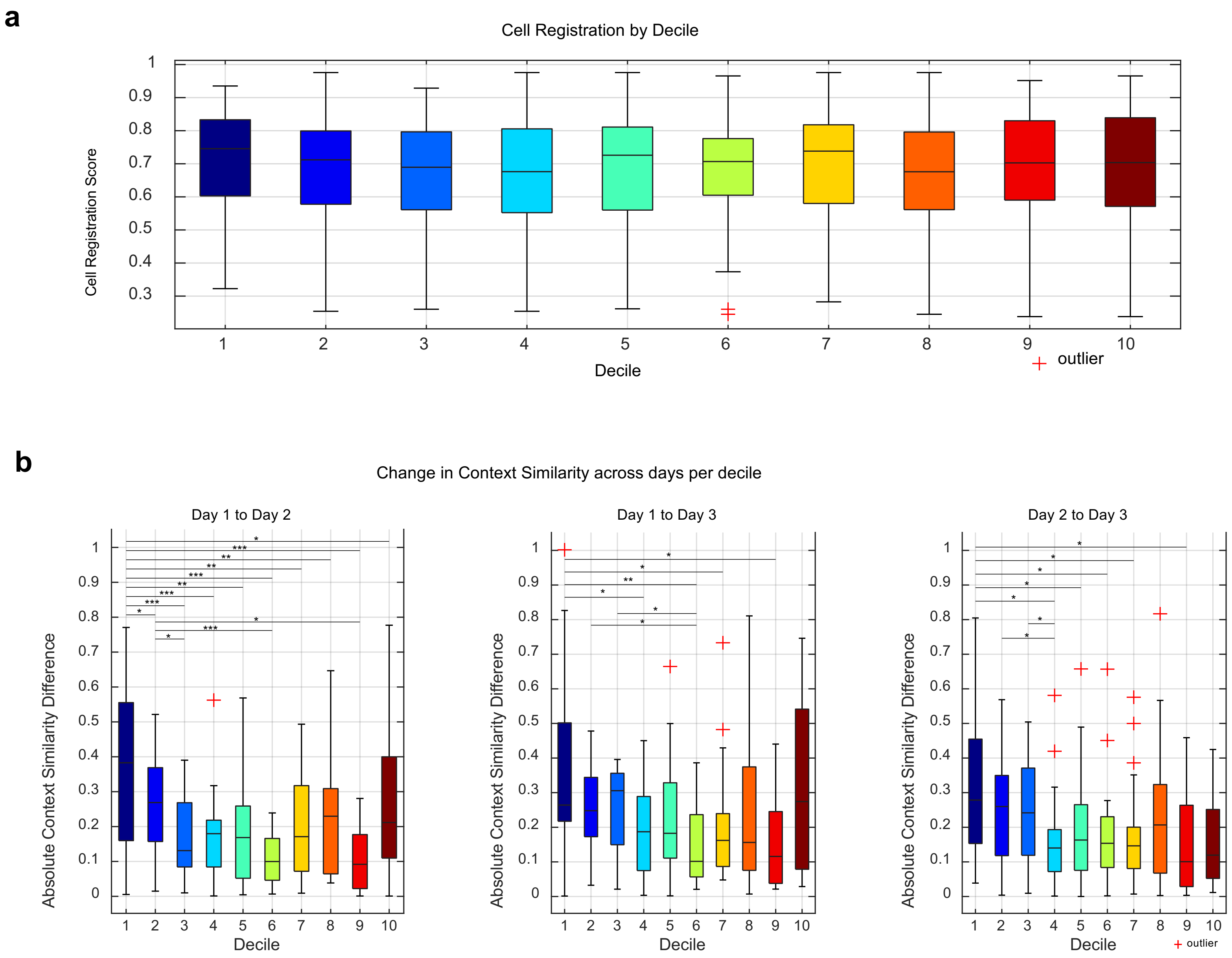

Supplementary Fig. 13. Complement of Fig. 6. **a)** Registration quality per decile. To determine if the registration quality influenced the remapping properties across context, we used the registration score index (range = 0–1) developed by Sheintuch et al. (2017), which is provided by the CellReg algorithm. This value evaluates the registration certainty of all cell-pairs within the population of registered cells by computing the footprint spatial correlations for each cell across days. Spatial registration quality was evaluated for any cell present during at least 2 days of testing by dividing the average correlation across context distribution in deciles. There were no significant differences in footprint quality across deciles [F(9, 1317) = 0.88, *p* = .54]. This indicates that changes in stability across time are not due to differences in spatial registration quality. **b)** Change in context similarity across days per decile. The absolute difference in correlations for all registered cells was calculated for each day pair by dividing the cells into the same deciles described in panel A. One way ANOVA results indicate that cells in decile 1 displayed more significant differences in similarity scores across context than cells from other deciles (Day 1 to Day 2: F(9,279) = 8.78, *p* < .0001; Day 1 to Day 3: F(9, 262) = 3.94, *p* < .0001; Day 2 to Day 3: F(9,226) = 3.93, *p* < .0001, Pos Hoc multiple comparisons shown in the graph). Registered cells present across deciles: *N*=236. In all Boxplots, the boxes indicate the upper and lower quartiles of the data and the whiskers (extending lines) the minimum and maximum outside the quartiles. The horizontal line indicates the median, and + indicates the presence of outliers. Number of registered cells: day 1 and 2: *N*=289, day 1 and 3: *N*=236; day 2 and 3: *N*=272. Asterisks represent *p* ≤ .05 (*), *p* < .01 (**) or *p* < .001 (***), # indicates a trend approaching the significance level set at .05. Source data are provided as a Source Data file.

Rate remapping estimating firing rate using calcium imaging recordings

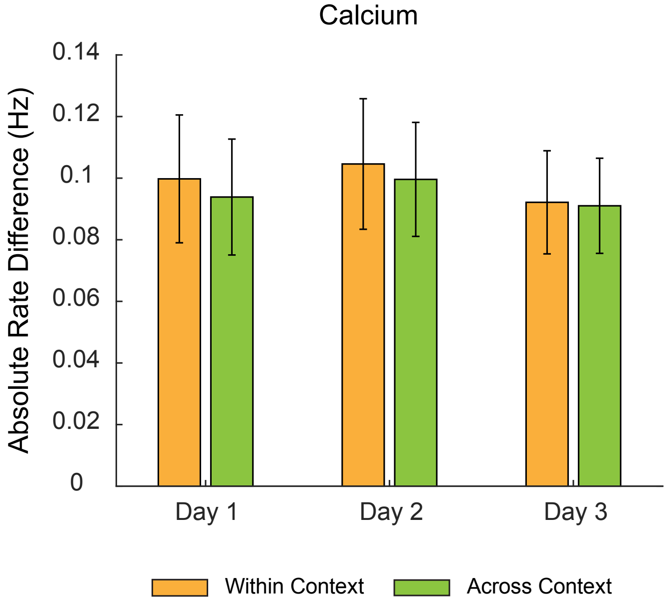

Supplementary Fig. 14. Complement of Figure 7. Bar charts showing rate differences using calcium traces. There were no differences within or across context on any day of training (p > .05 (paired t-tests, 2 tailed: day 1: $t(4)$ = 2.07, p =.11; day 2: $t(4)$ = 1.61, p = .18; day 3, $t(4)$ = 0.61, p = .58, N=5 mice). To calculate rate from calcium traces we applied CNMF-e to the recording videos of calcium activity, which returned associated inferred spike time series for each identified cell. We then filtered the data using our cell quality assessment describe din the main method section. We collected position data using DeepLabCut, which performed tracking using a trained mode. The positions were then transformed from pixel coordinates in the video frame to a canonical coordinate system with units of centimeters. From the difference of the animal position as a function of time we computed the speed of the animal in cm/sec. Because the calcium and behavior recording have difference sample times, the speed was interpolated into the sample times of the inferred spikes. To obtain the peak firing rate of each cell, we filtered out segments of the cell's inferred spikes where the animal was not moving (speed < 2 cm/sec), and then computed the maximum inferred spike magnitude which we divided by the median calcium frame rate to obtain a "peak firing rate" for the cell. It is worth noting that calcium traces are an indirect measure of spiking activity and do not have the temporal resolution of electrophysiological recordings. Bar chart represent mean ± standard error of the mean (SEM). Source data are provided as a Source Data file.

Rate remapping using mean and peak firing rate and different occupancy thresholds

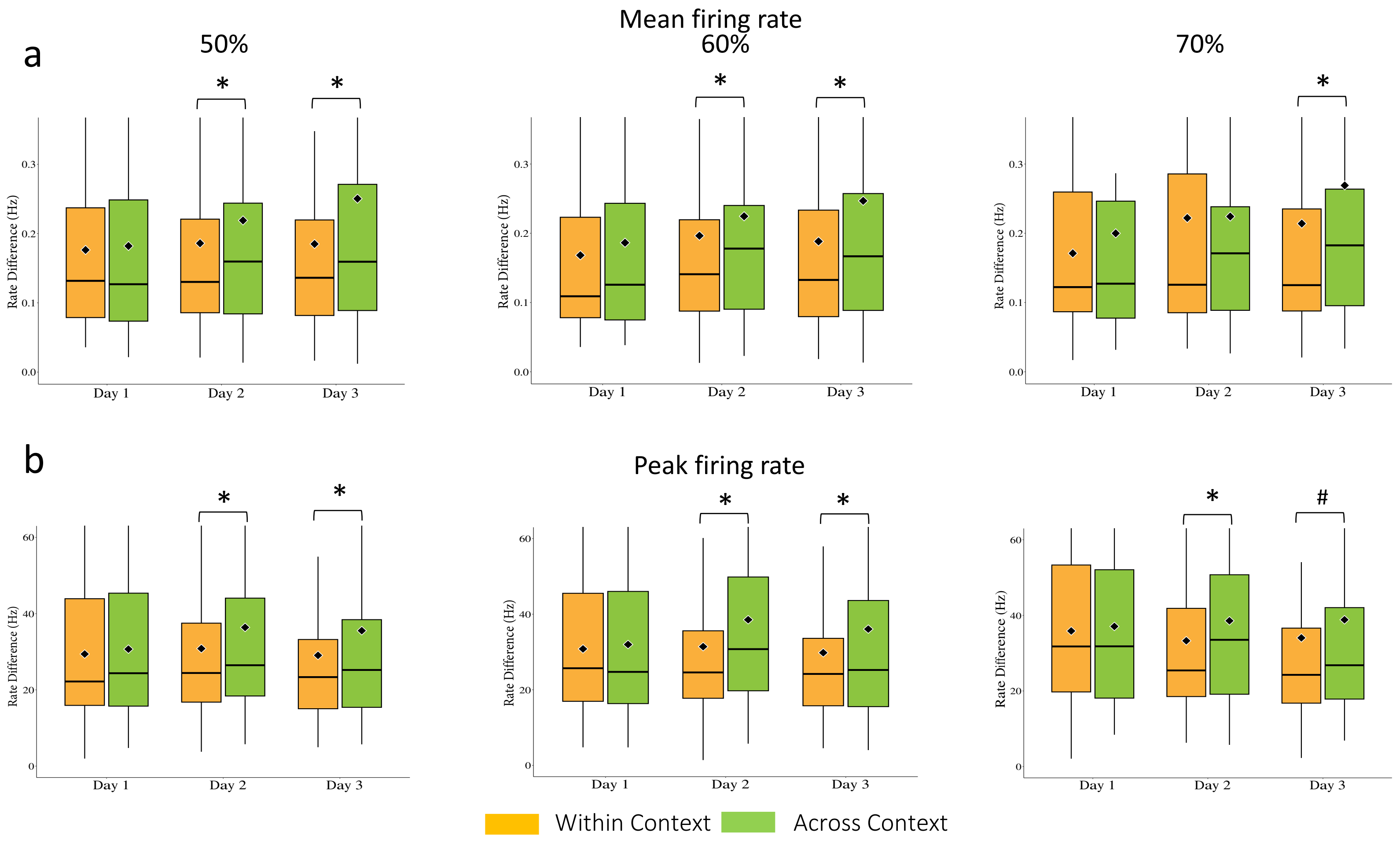

Supplementary Fig. 15. Complement of Fig. 7. Mean **(a)** and peak **(b)** firing rate changes within and across context provide similar differences using different occupancy thresholds, with the mean firing rate being slightly more consistent across conditions of occupancy. The results were equivalent across the variations of minimum occupancy (50%, 60%, and 70%) and measurement of rate differences (peak and mean firing rate). To compare the occupancy parameters, an analysis of effect sizes was performed. The Within-Across effect sizes were equivalent across occupancy and measurement conditions (day 1: p=0.97, N=80; day 2: p=0.16, N=80; day 3: p=0.99, N=69). All the graphs are Boxplots, in which the boxes indicate the upper and lower quartiles of the data and the whiskers (extending lines) the minimum and maximum outside the quartiles. The horizontal line indicates the median and the diamond the weighted mean used for statistics. Asterisks (*) represent a significance value set at .05. # indicates a trend approaching this significance level. Specific p levels and effect size are shown in Supplementary Table 8a. Source data are provided as a Source Data file.

Rate differences and rate predictions per cell type

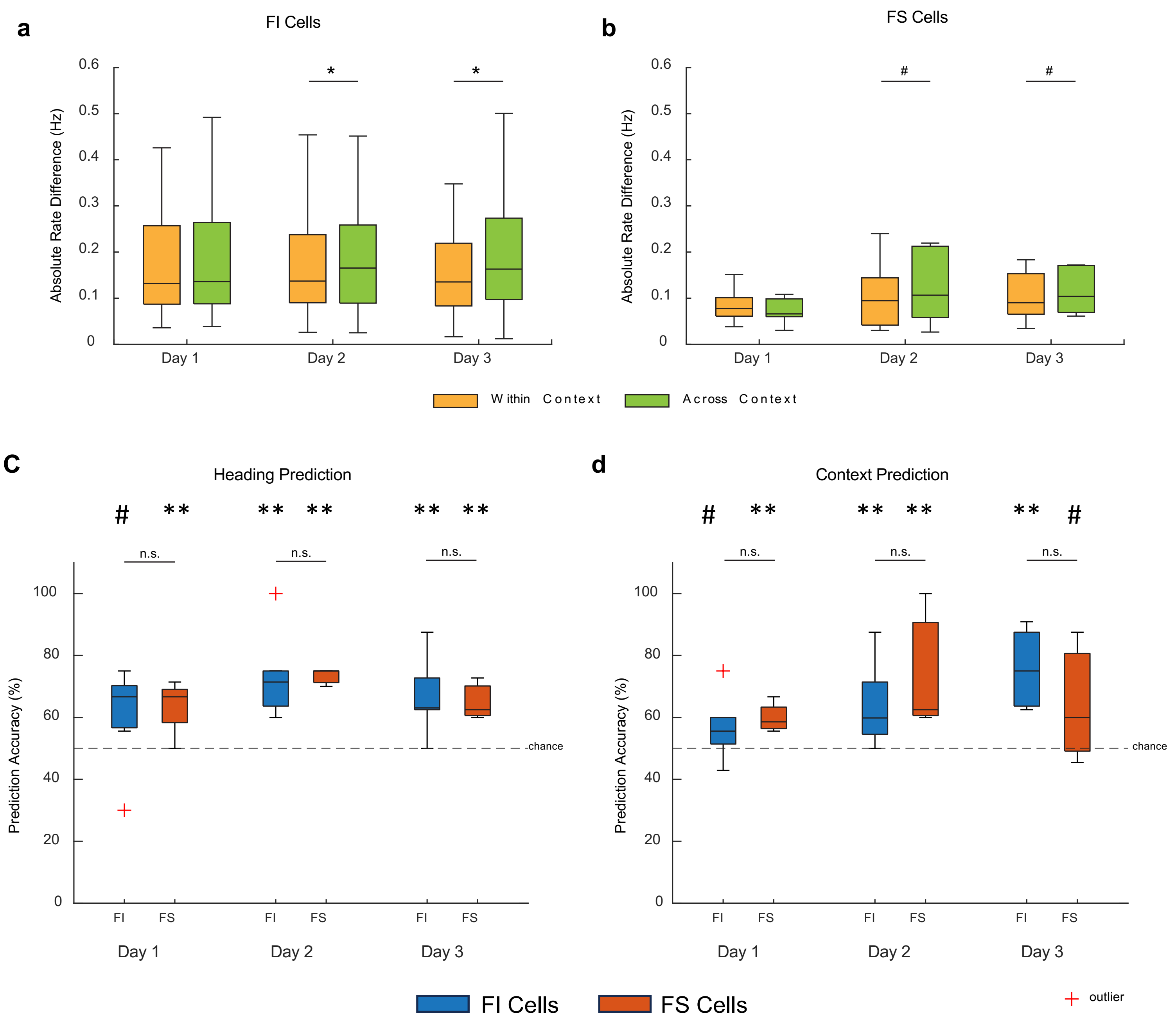

Supplementary Fig. 16. Complement of Fig. 7. **a-b)** Differences in mean firing rate within and across context in FI (a) and FS (b) cells. Robust repeated measures ANOVAS showed that FI displayed showed no differences on day 1 (p>.05), but significant differences on days 2 and 3 (p<.05). FS cells did not show significant differences on any day (p>.05). The absence of significant results in FS cells might stem from the limited yield of electrophysiology data. However, the trend in these cells is the same observed in FI cells (see Table S8B for p levels and effect size details). To strengthen these conclusions, an analysis of effect size was performed showing no rate differences between FS and FI cells (Table S8A). **c)** Heading prediction per cell type using rate. FI cells: Day 1: ξ = 1.31, p= . 0520; Day 2: ξ = 2.52, p< .0001; Day 3: ξ = 1.91, p<.0001. FS cells: Day 1: ξ = 0.94, p< .0001; Day 2: ξ = 5.19, p< .0001; Day 3: ξ = 1.44, p< .0001. **d)** Context prediction per cell type using rate. FI cells: Day 1: ξ = 0.89, p= .0640; Day 2: ξ = 0.93, p<.0001; Day 3: ξ = 1.52, p<.0001. FS cells: Day 1: ξ = 1.29, p<.0001; Day 2: ξ = 0.69, p<.0001; Day 3: ξ = 0.43, p= .0800. In both c and d, results are obtained using robust one-sample t-tests with respect to chance, with probability based on the xi (ξ) statistic. In addition, a robust t-test for dependent samples showed that there were no significant differences between FI and FS across days either for context or heading predictions (p >.05). These data suggest that rate changes integrate information about geometry and features. In all Boxplots the boxes indicate the upper and lower quartiles of the data and the whiskers (extending lines) the minimum and maximum outside the quartiles. The horizontal line indicates the median. Day 1: FI: 76 cells, FS: 8 cells; Day 2: FI: 68 cells, FS: 14 cells; Day 3: FI: 58 cells, FS: 11 cells. Asterisks represent p ≤ .05 (*), p ≤ .01 (**) or p ≤ .001 (***), # indicates a trend approaching the significance level we set at .05. Source data are provided as a Source Data file.

Analysis of effect size in rate remapping under distinct occupancy thresholds using mean or peak firing rate and in feature insensitive and feature-sensitive cells

a

		Mean Firing Rate		Peak Firing Rate	
Threshold	Day	*p* (within-across)	Effect size (ξ)	*p* (within-across)	Effect size (ξ)
50%	1	.533	0.02	.378	0.04
50%	2	.003	0.15	.008	0.15
50%	3	.005	0.15	.041	0.14
60%	1	.174	0.06	.628	0.02
60%	2	.039	0.12	.006	0.23
60%	3	.007	0.12	.020	0.15
70%	1	.495	0.05	.679	0.03
70%	2	.214	0.09	.010	0.20
70%	3	.036	0.16	.159	0.13

b

		Mean Firing Rate	
Cell Type	Day	*p* (within-across)	Effect size (ξ)
FI	1	.594	0.02
FI	2	.031	0.10
FI	3	.003	0.18
FS	1	. 317	0.11
FS	2	.085	0.23
FS	3	.102	0.12

Supplementary Table 8. Complement of Fig. 7, and Supplementary Fig. 15-16. Comparison of effect size for rate remapping within and across context throughout the days. **a)** Comparison of effect size using occupancy thresholds 50%, 60%, 70% for mean (left) and peak (right) firing rate differences for Supplementary Fig. 15a-b. **b)** Comparison of effect size of mean rate remapping for FS and FI cells for Supplementary Fig. 16a-b. FS=feature-sensitive; FI=feature-insensitive

References

1 Christopoulos, D.T. Roots, extrema and inflection points by using a proper Taylor regression procedure. *SSRN*. https://dx.doi.org/10.2139/ssrn.2521403 (2014).

2 Ritz, C., Baty, F., Streibig, J. C., and Gerhard, D. Dose-Response Analysis Using R. *PLOS ONE, 10*(12), e0146021 (2015)

3 Aristizabal, J. A., Ramos-Alvarez, M. M., Callejas-Aguilera, J. E. & Rosas, J. M. Attention to irrelevant contexts decreases as training increases: Evidence from eye-fixations in a human predictive learning task. *Behav Processes* **124**, 66-73, doi:10.1016/j.beproc.2015.12.008 (2016)

4 Martín-Guerrero, T. L., Rosas, J. M., Paredes-Olay, C. & Ramos-Alvarez, M. M. Psychophysical Curves for Tasting Based on A Dissociation Model. *J Sens Stud* **30**, 225-236, doi:10.1111/joss.12153 (2015).

5 Panik, M. J. 1 online resource (455 pages) (John Wiley & Sons, Inc.,, Hoboken, New Jersey, 2014).

6 Ahn, J. R. & Lee, I. Neural Correlates of Both Perception and Memory for Objects in the Rodent Perirhinal Cortex. *Cereb Cortex* **27**, 3856-3868, doi:10.1093/cercor/bhx093 (2017).

7 Pinheiro, J.C. and Bates, D.M. (2000) *Mixed-effects models in S and Splus*. Springer.

8 Climer, J.R., Dombeck, D.A. Information theoretic approaches to deciphering the neural code with functional fluorescence imaging*, eNeuro*.0266-21.2021.

9. Skaggs WE, McNaughton BL, Gothard KM (1993) An information theoretic approach to deciphering the hippocampal code 1030–1037. In: *Advances in neural information processing systems 5*, Hanson SJ, Cowan JD, Giles CL, eds). Burlington: Morgan-Kaufmann.
